# Mitochondria as Regulators of Nonapoptotic Cell Death in Cancer

**DOI:** 10.1002/mco2.70244

**Published:** 2025-07-23

**Authors:** Saloni Malla, Rabin Neupane, Saloni Sood, Noor Hussein, Mariam Abou‐Dahech, David Terrero, Charles R. Ashby, R. Jayachandra Babu, Amit K. Tiwari

**Affiliations:** ^1^ Department of Pharmacology and Experimental Therapeutics College of Pharmacy and Pharmaceutical Sciences University of Toledo Toledo Ohio USA; ^2^ Department of Pharmaceutical Sciences College of Pharmacy University of Arkansas for Medical Sciences Little Rock Arkansas USA; ^3^ Department of Pharmaceutical Sciences College of Pharmacy St. John's University Queens New York USA; ^4^ Department of Drug Discovery & Development Harrison School of Pharmacy Auburn University Auburn Alabama USA

**Keywords:** autophagy, cancer, ferroptosis, mitochondria, necroptosis, nonapoptotic cell death

## Abstract

Mitochondria are involved in cell survival and metabolic processes including adenosine triphosphate production, heme biosynthesis, reactive oxygen species, and iron and calcium homeostasis. Although mitochondria are well known to contribute to apoptosis, a growing body of evidence indicates that mitochondria modulate nonapoptotic cell death (NACD) mechanisms, including autophagy, necroptosis, ferroptosis, paraptosis, pyroptosis, parthanatosis, and cuproptosis. These NACD pathways differ in molecular triggers, morphological characteristics, and immunological consequences, but they all involve mitochondria. For example, mitochondrial ROS and lipid peroxidation play a role in ferroptosis, whereas mitochondrial depolarization and the release of apoptosis inducing factor are paramount to parthanatosis. Mitochondrial swelling is a hallmark of paraptosis, whereas mitochondrial disruption is associated with pyroptosis. Autophagy, though primarily a survival mechanism, is also regulated by mitochondrial dynamics in cancer cells. In cuproptosis, mitochondrial protein aggregates when iron–sulfur cluster proteins are disrupted, resulting in copper‐dependent cell death. There are many factors that influence NACD, including mitochondrial membrane potential, bioenergetics, calcium flux, metabolites, and interactions with the endoplasmic reticulum. The review comprehensively summarizes our understanding of mitochondrial and NACD interactions, particularly in cells resistant to classical apoptosis agents. Therapeutic vulnerabilities associated with mitochondria‐mediated NACD could lead to next‐generation therapies.

## Graphical abstract

Mitochondrial fission and fusion are key processes in maintaining cellular health. Fission is driven by proteins like, Fis‐1, which recruits DRP‐1, to facilitate the division of mitochondria. Fusion, however, is mediated by mitofusion 1(MFN1), mitofusion 2(MFN2), and optic atrophy 1 (OPA1), which work together to merge mitochondria, allowing functional maintenance. These opposing but complementary processes are crucial for cellular adaptation and energy regulation.

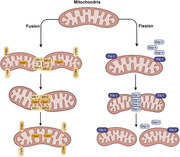

## 1 Introduction

**FIGURE 1:**
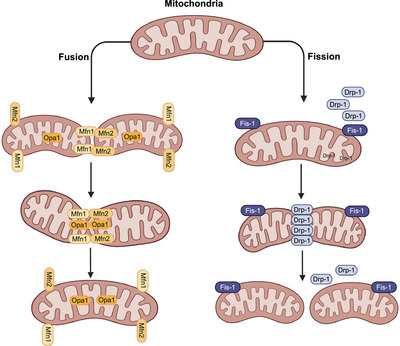
Mitochondrial dynamics. Mitochondria undergoes a dynamic equilibrium of fission and fusion. In mitochondrial fusion, two or more mitochondria merge to form a single, larger mitochondrion. The outer mitochondrial membrane proteins mitofusin 1 (MFN1) and mitofusin 2 (MFN2), as well as the inner mitochondrial membrane protein optic atrophy 1 (OPA1), are essential for mitochondrial fusion. On the other hand, in mitochondrial fission, a single mitochondrion divides into two or more smaller mitochondria. The cytosolic dynamin‐related protein 1 (DRP1) initiates mitochondrial fission by translocating to the outer mitochondrial membrane. Mitochondrial fission 1 protein (FIS1), located on the outer mitochondrial membrane, then recruits DRP1 to the mitochondria, facilitating the fission process. The figure created with BioRender.com.

The mitochondria are double‐membraned organelles found in eukaryotic cells and are often referred to as the powerhouses of the cell [1]. A major function of mitochondria is to produce ATP by oxidative phosphorylation (OXPHOS) [2, 3]. However, numerous studies have reported that a variety of cellular processes are regulated by mitochondria, including energy metabolism, redox homeostasis, calcium signaling, iron metabolism, innate immunity, β‐oxidation of fatty acids, and programmed cell death, that is, apoptosis [4, 5, 6, 7, 8, 9]. Mitochondria are highly dynamic organelles that modify their overall morphology, mass, and cellular location, in response to bioenergetic or oxidative stress, by activating separate molecular pathways that regulate fission, fusion, mitophagy, mitochondrial biogenesis, and motility [10, 11]. Mitochondrial morphology (i.e., shape, size, number, and network) is regulated by the division of mitochondria into smaller mitochondria through a fission process or the physical merging of two mitochondria through mitochondrial fusion process. The dynamic processes of fission and fusion maintains the integrity and function of mitochondria by allowing the exchange of intramitochondrial content, such as mitochondrial DNA (mtDNA), nucleoids, lipid membranes, metabolites, and substrates [12, 13]. In mammalian cells, mitochondrial fission and fusion are closely regulated by a complex protein assembly, consisting of mitochondrial fission proteins: dynamin 1 like (DNM1L, commonly known as DRP1), mitochondrial fission factor, human fission factor‐1, mitochondrial elongation factor ½ (also known as Mid51/49), and mitochondrial fusion proteins: mitofusin 1 (MFN1), mitofusin 2 (MFN2), and optic atrophy protein 1 (OPA1) (Figure 1) [14, 15].

Mitochondrial biogenesis (i.e., the synthesis of mtDNA, membrane and protein, which increases mitochondrial size and number) and mitochondrial turnover/biodegradation through mitophagy (mitochondrial autophagy) regulate cellular adaptation in response to mitochondrial dysfunction [16]. Consequently, both processes, which are highly regulated, significantly affect mitochondrial and cellular homeostasis. Furthermore, the balance between mitochondrial production and degradation controls variations in the mitochondrial mass the mitochondrial population, in terms of quantity and quality and mitochondrial content [17, 18, 19]. Similarly, the subcellular localization of mitochondria vary, depending on the level or type of cellular stress, such as hypoxia‐induced perinuclear clustering of mitochondria [20], microtubule‐mediated mitochondrial movement toward lysosomes, as in mitophagy [21], and presynaptic and postsynaptic terminals in neurons [22, 23, 24, 25].

A key objective of this review is to highlight the role of mitochondria in nonapoptotic cell death (NACD). A comprehensive analysis of the role of mitochondria in regulating NACDs such as necroptosis, autophagy, ferroptosis, pyroptosis, paraptosis, parthanatos, and cuproptosis is provided. Regulatory mechanisms for NACD are emerging concepts with potential applications in cancer biology and therapeutics. Frequently, NACD processes are regulated processes, but they lack typical inducers, signaling cascades, biochemical changes, or even morphological changes that differentiate them from apoptosis. Nonetheless mitochondria play a crucial role in many cases, and changes in mitochondrial dynamics, morphology, and/or modulation of key fission and fusion proteins may represent therapeutic targets in cancer. Here, we further investigated how membrane proteins, lipids, mitochondrial reactive oxygen species (mtROS), bioenergetics, mitochondrial permeability transition (MPT), and endoplasmic reticulum (ER) interact to initiate and regulate NACD pathways.

## 2 Cross‐Regulation of Mitochondrial Dynamics in NACD

Mitochondrial fission and fusion processes are tightly regulated by a set of proteins [26, 27, 28]. In apoptotic cell death, mitochondrial fragmentation is primarily facilitated by the GTPase DRP1 and is closely linked to mitochondrial outer membrane permeabilization [29]. Recent studies have demonstrated that mitochondrial fusion and fission proteins also play central roles in orchestrating NACD pathways, particularly ferroptosis and necroptosis [30]. Mitochondrial integrity plays an important role in ferroptosis [31]. The Inhibition of Drp1, either through dominant‐negative mutants or siRNA‐mediated knockdown, has been shown to effectively suppress mitochondrial fission, resulting in elongated mitochondrial networks. Conversely, overexpression of DRP1 induces mitochondrial fragmentation, indicating its essential role in mediating fission [26]. Disruption of the mitochondrial fission machinery, through DRP1 knockdown or depletion, significantly delays the execution of ferroptosis, highlighting a crucial role forDRP1 [32]. In addition to Drp1, other mitochondrial regulators, such as the fusion proteins MFN1, and MFN2, also contribute to ferroptosis. The STING1–MFN complex promotes outer membrane fusion and facilitates crosstalk between the ER and mitochondria, increasing mitochondrial ROS production an facilitating ferroptosis progression. This mechanism operates independently of mitophagy and highlights a fusion facilitated route to ferroptosis, that complements the well‐established role of Drp1‐mediated fission [33, 34]. Mitochondrial proteins, such asDRP1, also play an important role in regulating cell fate during oxidative stress. Guo et al. [55] found that DRP1 stabilizes p53 and is necessary for its movement to the mitochondria under stress conditions. Their study showed that when Drp1 binds to p53, it produces to mitochondria‐related necrotic cell death. Mitochondrial fusion, fission, and autophagy, work together, as a quality control system, to maintain mitochondrial bioenergetics [35]. In melanoma cells, mitochondrial dynamics and mitophagy have been shown to influence regulated cell death, that is critical in the response to a hypoxia‐inducible factor (HIF) inhibitor, BAY 87–2243. Knockdown of ATG5, a key autophagy protein, suppressed BAY‐induced autophagosome formation, decreases ROS accumulation, and prevented cell death, indicating autophagy's necessary role in mediating this response. Furthermore, DRP1 knockdown caused mitochondrial filamentation with a marked attenuation of BAY‐induced cell death [36, 37]. Similarly, PTEN‐induced kinase 1 (PINK1)–phosphorylated MFN2 initiates autophagosome formation and subsequent lysosomal degradation, by acting as a receptor for Parkinson's disease protein 2 (Parkin/PARK2), producing to the ubiquitination of OMM proteins such as voltage‐dependent anion channel (VDAC), MFN1, and MFN2, followed by the recruitment of sequestosome 1 (SQSTM1, a cargo receptor for the degradation of ubiquitinated proteins). SQSTM1 interacts with microtubule‐associated protein light chain 3 (LC3B), producing mitochondrial sequestration by the phagophore [38, 39, 40]. However, it has also been reported that SQSTM1 is involved in the clustering of mitochondria after recruitment by PARK2, but it is not essential for mitophagy induction [41].

Mitochondrial fission has also been implicated in necroptosis. During this process, the receptor‐interacting serine/threonine protein kinase 1 (RIPK1) increases Drp1 activity by catalysing its phosphorylation at Ser616, a process that relies on receptor‐interacting serine/threonine protein kinase 3 (RIPK3) but does not involve the mixed lineage kinase domain‐like protein (MLKL). RIPK1 and RIPK3 modulate mitochondrial fission and ROS generation by dephosphorylating Drp1 at Ser637 and phosphorylating it at Ser616 [42]. Drp1 induces mitochondrial fragmentation, causing increased mitochondrial dysfunction, and resulting in the production of ROS, and the opening of the mPTP. These mitochondrial changes can amplify necroptotic signaling and contribute to cell death [43]. Alternatively, the short form of mitochondrial serine/threonine protein phosphoglycerate mutase family 5 (PGAM5S) induces DRP1 activation, through dephosphorylation at Ser637, induce mitochondrial fission and ultimately, necroptosis [44, 45]. The inactivation of PGAM5S significantly decreases DRP1 activity and the genetic knockdown of DRP1 and pharmacological inhibition with mdivi‐1 (a DRP1 inhibitor) at higher concentrations, of necroptotic cell death [44]. However, the role of PGAM5 and DRP1 in necroptosis is context‐dependent, as knockdown of PGAM5 or DRP1 failed to prevent necroptosis in NIH3T3 cells, following TNF or RIPK3 activation [46]. This suggests that although DRP1‐mediated mitochondrial fission contributes to necroptosis in specific contexts, it may not be universally required.

Together, these findings highlight the multifaceted roles of mitochondrial fission and fusion proteins in regulating NACD.

## 3 Mitochondria in Necroptosis Regulation

Necroptosis is a programmed, regulated, caspase‐independent, necrotic cell death, that is initiated by the activation of death receptors, such as tumor necrosis factor receptor superfamily member 6 (Fas) [47], tumor necrosis factor receptors 1 and 2 (TNFR1 and TNFR2), TNF‐related apoptosis‐inducing ligand receptors TRAILR1 and TRAILR2 [48], pattern recognition receptors, that include toll‐like receptors TLR3 and TLR4 [49, 50], the Z‐DNA binding protein 1 (also known as DAI) [49, 51, 52], cytosolic nucleic acid sensors, such as retinoic acid‐inducible gene I and transmembrane protein 173 (also known as stimulator of interferon genes, (*STING*), and adhesion molecules (such as ICAM1, VCAM1, and E‐selectin) [53, 54, 55]. Ligand binding to death receptors on the plasma membrane induces their clustering and assembly of cytosolic macromolecular complexes, which, depending on the cellular state, induces either NF‐kB signaling and proliferation, apoptosis, if NF‐kB signaling is blocked, or necroptosis, if apoptosis is blocked. Signaling is mediated by adaptor proteins recruited to ligand‐death receptor complexes, including Fas‐associated via death domain (FADD) for FasL–Fas complex, TNFRSF1A‐associated via DD for TNF–TNFR complexes, or TLR3/4 receptor‐interacting factor for TNFR and RIPK1 [56]. Phosphorylated RIPK1 activates RIPK3, in the absence of caspase‐8 activation, by its homotypic RIP homology interaction motif, resulting in the formation of the necrosome signaling complex [57, 58, 59]. In this complex, RIPK3 is autophosphorylated and this recruits and activates MLKL via its phosphorylated residues [60, 61]. This results in MLKL oligomerization, which destabilizes and compromises the plasma membrane integrity, producing necroptotic cell death [62, 63, 64, 65]. This type of cell death is characterized by cellular and organelle swelling, the absence of nuclear fragmentation, mitochondrial dysfunction, plasma membrane disruption, and the release of proinflammatory intracellular components [66].

**FIGURE 2:**
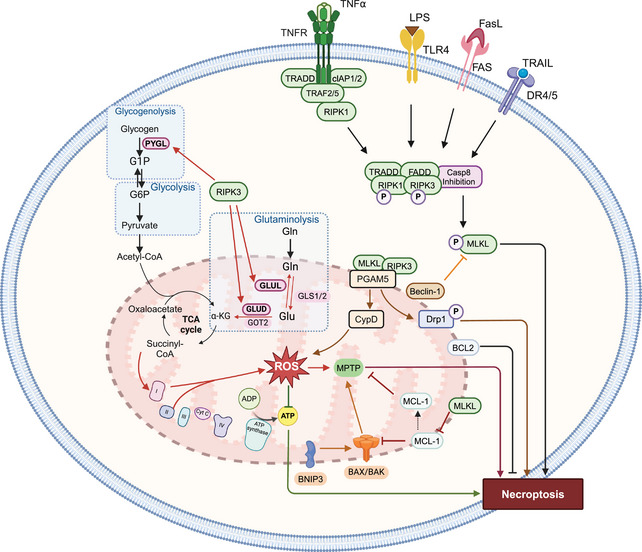
The role of mitochondria in necroptosis. RIPK3 and MLKL interact with mitochondrial serine/threonine protein phosphatase (PGAM5), which activates of cyclophilin‐D (Cyp‐D). Cyp‐D further increases ROS accumulation that leads to the opening of mitochondrial permeability transition pores (MPTP) and a decrease in ATP production, leading to necroptosis. RIPK3 regulates certain energy metabolism enzymes, such as glycogen phosphorylase (PYGL), glutamate‐ammonia ligase (GLUL), and glutamate dehydrogenase 1 (GLUD1), resulting in ETC complex I and II‐derived mtROS production. The proapoptotic proteins, BAX, BAK, and BH3‐only proteins (BMF, BNIP3) positively regulate necroptosis. Antiapoptotic proteins, such asBCL‐2, MCL‐1, BH3‐only proteins (e.g., BECLIN‐1), negatively regulate necroptosis. The figure created with BioRender.com.

It has been hypothesized that mitochondria may be regulators of necroptosis, based on increase or accumulation of ROS and MPT [67, 68]. During necroptosis, mitochondria undergo ultrastructural alterations. For example, TNF‐α produces enlarged mitochondria with clear matrix spaces and a lower number of cristae, in mouse fibrosarcoma WEHI‐164 cells [69]. Similarly, TNF‐induced necroptosis in L929 mouse fibroblast cells produces mitochondrial degradation [70]. The early development of onion‐like structures from vesiculation and the clustering of mitochondrial cristae inside the mitochondrial matrix are characteristic of cells undergoing necroptosis [70]. The cristae develop into rounded, electron‐dense projections that protrude into the mitochondrial matrix space [70]. Long‐term exposure of L929 cells to TNF causes a prominent, multilamellar vesicle of clustered cristae to form inside mitochondria [70]. The mitochondria of cells undergoing necroptosis are large and swollen with disoriented cristae that are separated from the  IMM [70, 71, 72]. Swollen mitochondria and mitochondrial damage occurred in the presence of second mitochondria‐derived activator of caspase (Smac) mimetic and carbobenzoxy‐valyl‐alanyl‐aspartyl‐[O‐methyl]‐fluoromethylketone (Z‐VAD‐FMK) in HT‐29 cells [73], TNF‐α and zVAD.fmk in mouse embryonic fibroblasts (MEFs) [74], incubation of A2780 cells with aldehyde dehydrogenase 1 family‐specific inhibitor (673A) [75], and shikonin in HL60 cells [76]. However, there are also data suggesting that mitochondria are not involved in necroptosis [77]. The role of mitochondria in regulating necroptosis is summarized in Figure 2.

### 3.1 Mitochondrial Homeostasis in Necroptosis Induction

Studies have shown that the MPT pores (MPTPs) are involved in the regulation of necroptosis by mitochondria [78, 79, 80]. MPTP is an IMM transmembrane protein that opens in response to ROS and Ca^2+^ overload in the mitochondrial matrix, leading to the entry of molecules >1.5 kDa into mitochondria [81]. This abrupt increase in IMM permeability, also known as MPT, causes mitochondrial osmotic swelling, alterations in mitochondrial transmembrane potential (ΔΨM), impairment of OXPHOS, accumulation of ROS, and eventually mitochondrial rupture [82, 83, 84]. The inhibition of MPT by the compound, S‐15176, significantly decreased TNF‐α‐induced necroptotic cell death in microvascular endothelial cells (MVECs) [79]. Cyclophilin‐D (Cyp‐D) regulates MPTP opening [85, 86, 87, 88] and studies suggest that Cyp‐D plays a role in necroptosis [74, 78, 79, 89]. Diarachidonoylphosphoethanolamine induced necroptosis and necrosis in malignant pleural mesothelioma cells mediated by: (1) RIPK1 and (2) Cyp‐D‐mediated opening of MPTP and a subsequent decrease in ATP levels [78]. In Cyp‐D‐deficient MEFs, there were no ultrastructural changes typical of necroptotic cell death induced by TNF‐α and zVAD‐fmk in wild MEFs and these cells were partially resistant to TNF‐α‐ and zVAD‐fmk‐induced necroptotic cell death [74]. Furthermore, Cyp‐D‐deficient MEFs were resistant to caerulein‐mediated necroptotic cell death [74, 79]. Similarly, the inhibition of the opening of MPTP by cyclosporin A, a Cyp‐D and calcineurin inhibitor, decreased TNF‐α‐mediated necroptotic cell death, whereas necroptotic death occurred after incubation with FK506, a non‐Cyp‐D binding and calcineurin inhibitor [79]. Cyp‐D‐deficient MVECs (obtained from Cyp‐D‐deficient mice) and Cyp‐D inactivated MVECs, TNF‐α did not produce necroptosis, suggesting that Cyp‐D is involved in mediating Cyp‐D necroptotic death [79]. RIPK3‐induced necroptosis in endothelial cells was accompanied by MPTP opening and an increase in the phosphorylation of Ser31 in Cyp‐D [80]. However, Cyp‐D deletion did not rescue embryonic lethality in caspase‐8‐deficient mouse, suggesting that necroptosis may not occur through MPT [46].

In contrast, bromocriptine‐induced RIPK3‐mediated necroptosis in MMQ prolactinoma cells increased the levels of the phosphorylated form of Cyp‐D and PGAM5, and the levels of these phosphorylated proteins were significantly decreased after incubation with Necrostatin‐1, an inhibitor of necroptosis [89]. PGAM5 is an IMM protein that regulates mitochondrial dynamics and programmed/regulated cell death, due to its phosphatase activity [45]. Furthermore, the knockdown of genes for Cyp‐D or PGAM significantly decreased bromocriptine‐induced necroptosis in MMQ cells that was characterized by a decrease in cellular ROS levels and an increase in ATP levels [89]. PGAM5L (the long splice variant of PGAM5) is the kinase substrate of RIPK1/RIPK3, which activates and phosphorylates PGAM‐S on mitochondria [44]. Inactivating either PGAM5L or PGAMS in HeLa or HT‐29 cells inhibited TNF‐α–Smac mimetic–z‐VAD‐fmk‐induced necroptotic cell death [44]. However, there are studies suggesting that PGAM5 do not mediate the execution of necroptosis. TNF‐induced necroptosis in mice was not prevented in PGAM5‐deficient mice, which might be attributed to the expression of PGAM5‐L but not PGAM5‐S [90].

Alternatively, melatonin inhibited RIPK3‐mediated necroptosis in endothelial cells by inhibiting Cyp‐D phosphorylation and decreasing PGAM5 levels [80]. Similar to the effects of melatonin, PGAM5 knockdown in cardiac microvascular endothelial cells (CMECs) inhibited necroptosis by decreasing Cyp‐D phosphorylation and reversing the mitochondrial membrane potential (MMP), which is an indirect marker of the opening of MPTPs [80]. Similarly, lipopolysaccharide (LPS)‐activated necroptotic cell death in cardiomyocytes involved the activation of the RIPK3/PGAM5 signaling pathway [91]. Thus, necroptosis initiated by RIPK3 activates PGAM5, which increases Cyp‐D phosphorylation and MPTP opening, increasing ROS accumulation, which ultimately produced to cell death [80, 91]. Interestingly, the MLKL inhibitor, GW806742X [92], inhibited Cyp‐D‐mediated necroptosis, indicating that MLKL activity regulates Cyp‐D‐mediated necroptosis. [79]. Furthermore, the translocation of RIP3‐downstream MLKL from the cytosol to mitochondria occurred in TNF‐α and zVAD‐induced necroptotic cell death in MEFs but was absent in RIPK3‐deleted MEFs [74]. Although only a few studies support the hypothesis that mitochondria have no function in necroptosis, the majority suggested that the RIPK1–RIPK3–MLKL–PGAM5–Cyp‐D–MPTP–DRP1 axis was a putative mitochondrial mechanism for necroptosis execution.

### 3.2 Mitochondria‐Derived ROS Regulates Necroptosis

There is evidence that mtROS are involved in the regulation of necroptosis [70, 93]. For example, necroptosis initiated by TNF‐α alone or in combination with BV6 (a Smac mimetic) involved the accumulation of intracellular ROS in mouse fibrosarcoma cells, mouse fibroblasts, and FADD‐deficient acute lymphoblastic leukemia [93, 94, 95, 96, 97]. ROS scavengers, such as butylated hydroxyanisole (BHA), N‐acetylcysteine (NAC), α‐tocopherol, ethyl pyruvate, and amytal, an inhibitor of ETC [98], significantly inhibited necroptosis induced by TNF‐α alone or in combination with BV6 in Jurkat cells, MV4‐11 cells, L929 cells, HeLa cells overexpressing RIP2, U937 cells, and primary peritoneal macrophages, MEFs in combination with dexamethasone, in acute myeloid leukemia cells (Tanoue and Jurkat cells) [93, 94, 96, 97, 99, 100]. In contrast, in HT‐29 cells, neither BHA nor amytal significantly affected necroptosis induced by TNF‐α, zVAD, and the Smac mimetic, compound 3 [73, 101]. Another study reported that mitochondrial depletion prevented necroptosis‐induced ROS generation but not TNF‐α‐ and zVAD‐induced necroptosis, in 3T3‐SA and murine peripheral lymph node endothelial cells [46]. In contrast, mitochondrial depletion in L929 cells decreased necroptotic cell death induced by TNF‐α [99]. Thus, the results of these above mentioned studies suggests that the regulation of necroptosis by ROS in cells is context dependent [46, 99].

TNF‐α‐mediated cell death was reported to be regulated by mitochondrial but not cytosolic ROS, in L929 and RAW 264.7 cells, which was confirmed by the inhibition of ROS levels by 2‐thenoyltrifluoroacetone, a mitochondrial respiratory chain complex II inhibitor but not by diphenyleneiodonium, an inhibitor of nicotinamide adenine dinucleotide phosphate (NADPH) oxidase [102].

Similarly, BAY 87–2243, an inhibitor of mitochondrial complex I [103], induced necroptotic and ferroptotic cell death, by increasing cellular ROS and lipid peroxide levels [104]. Furthermore, shikonin‐induced necroptosis in SHG‐44 glioma cells was due, in part, to high levels of superoxide in mitochondria after MLKL activation, which significantly disrupted or decreased the MMP, producing mitochondrial dysfunction [105]. The inhibition of the subunit of mitochondrial complex I (NADH dehydrogenase (ubiquinone) 1 beta subcomplex 8, NDUFB8) significantly decrease cell death induced by TNF‐α alone or in combination with BV6, in L929 cells [97]. Furthermore, mtROS levels regulate RIP1 by targeting three cysteines in RIP1 at positions 257, 268, and 586, and catalyze the autophosphorylation of Ser161, resulting in the recruitment of RIP3 into the necrosome, producing necroptosis [99]. TNF‐α‐mediated ROS generation and necroptosis are dependent on RIPK3 [106]. Alterations in RIPK3 levels produced proportional changes ROS levels caused by TNF‐α plus zVAD [107]. Thus, mtROS targets downstream RIP1 oligomerization and upstream from RIP3 in TNF‐α‐mediated necroptosis [99]. Furthermore, ROS production occurs downstream of glucose metabolism in the mitochondria from the ETC and therefore, increases in glucose concentration increase ROS levels [108]. In hyperglycemic conditions, TNF‐α and cycloheximide (CHX, an inhibitor of protein synthesis [109])‐induced cell death changed from apoptosis to necroptosis, which occurred in the presence of increased mtROS and RIP1 phosphorylation and the loss of caspase‐3, caspase‐6, and caspase‐9 [108, 110]. The suppression of glycolysis by 2‐deoxyglucose inhibited hyperglycemia‐induced TNF‐α/CHX‐induced necroptotic cell death [108]. Interestingly, the inactivation or pharmacological inhibition of enzymes involved in energy metabolism, such as glycogen phosphorylase (PYGL), glutamate‐ammonia ligase (GLUL), and glutamate dehydrogenase 1 (GLUD1), inhibited ROS production caused by TNF plus zVAD, which produced cell death [106]. Overall, the results suggest that energy metabolism and ETC‐associated ROS generation regulates RIPK1 and RIPK3 activity in necroptotic cell death.

PARP1 (poly (ADP‐ribose) polymerase activation has also been implicated in oxidative stress and necroptosis [70, 111]. The overactivation of PARP‐1 has been reported to produce dysfunction of mitochondrial respiratory chain complex I and an increase in ROS production [112]. The inhibition of PARP‐1 by *O*‐(3‐piperidino‐2‐hydroxy‐1‐propyl)nicotinic amidoxime (BGP‐15) maintained the ΔΨM and mitochondrial respiration and decreased ROS levels [113, 114, 115]. Glutamate‐induced necroptosis occurred in the presence of increased ROS levels and PARP‐1 activation and incubation with the PARP‐1 and PARP‐2 inhibitor, PJ34 [116] prevented glutamate‐induced necroptosis in HT‐22 cells [117, 118]. Thus, inhibiting PARP‐1 may decrease the likelihood of necroptosis by preventing ROS formation and preserving mitochondrial function. In contrast, TRAIL‐induced necroptosis in HT‐29 cells was regulated by RIPK1/RIPK3‐dependent PARP‐1 activation; however, ROS production was not involved in TRAIL‐mediated necroptotic cell death.

### 3.3 B‐Cell Lymphoma 2 Protein Family Mediated Necroptosis

There are data suggesting that the prodeath B‐cell lymphoma 2 (Bcl‐2) proteins, located in the mitochondrial outer membrane, contribute to necroptotic cell death [119, 120, 121]. The Bcl‐2 family is comprised of the proapoptotic pore‐forming proteins (BAX, BAK, Bcl‐2 related ovarian killer), antiapoptotic proteins (Bcl‐2, Bcl‐W, B‐cell lymphoma‐extra‐large (BCL‐X_L_), myeloid leukemia‐1 (MCL‐1), Bcl‐2‐related protein A1 (Bcl‐2A1 or BFL‐1), and the proapoptotic BH3‐only proteins (BMF), Bcl‐2‐associated death promoter (BAD), BH3‐interacting domain death agonist (BID) Bcl‐2‐interacting killer, Bcl‐2 interacting mediator of cell death, NOXA (Latin for damage), p53 upregulated modulator of apoptosis, Harakiri Bcl‐2 interacting protein, and Mcl‐1 ubiquitin ligase E3 (MULE, also known as BCL‐G) [122, 123]. Bcl‐2 interacting protein 3 (BNIP3), breast cancer 2, apolipoprtein6, microtubule‐associated protein 1, and BECLIN‐1, are proteins that contain other BH3‐domains but their exact role as prodeath molecules remains to determined [122, 124].

The *BMF* gene was identified as a regulator of necroptosis induced by zVAD.fmk and TNF‐α *BMF* silencing protected NIH 3T3 cells from TNF‐α/CHX‐induced necroptosis [125]. Similarly, the proapoptotic proteins, BAX and BAK, are involved in necroptosis [74, 126]. Green tea polyphenol (GTP) activated BAX and BAK interdependently, followed by mitochondrial translocation and subsequent cytochrome *c* release, to induce necroptosis in p53‐deficient Hep3B cells [126]. The overexpress of BCL‐2 prevented the GTP‐induced necroptosis of Hep3B cells, whereas the knockdown of *BAX* and *BAK* significantly decreased cytochrome *c* release and necroptotic cell death in Hep3B cells [126]. The knockout of BAX and BAK produced resistance to TNF‐α‐ and zVAD‐fmk‐induced necroptosis in MEFs [74]. Furthermore, Smac mimetic (BV6)/glucocorticoid (dexamethasone)‐induced necroptosis involves BAK activation by RIP3, which was followed by MLKL‐mediated ROS generation, that ultimately resulted in mitochondrial dysfunction and the death of acute myeloid leukemia cells (Tanoue and Jurkat cells) and colon carcinoma cells (MV4‐11 and HT‐29 cells), which was confirmed by a significantly decrease in necroptosis after Bak knockdown [100]. It has been reported that BCL‐2 and the adenovirus E1B 19‐kDa‐interacting protein 3 (BNIP3) play a role in necroptosis [127, 128]. BNIP3 has been shown to mediate MPT and mitochondrial dysfunction, by activating BAX and BAK in MEFs [129, 130]. Necroptosis facilitated by TNF‐α increases ROS generation and the insertion of BNIP3 in the mitochondria of A549 lung adenocarcinoma cells [127]. TNF‐α increases the levels of BNIP3 and its expression level alters the susceptibility of A549 cells to TNF‐α‐induced necroptosis [127]. The mitochondrial matrix isoform of another BCL‐2 protein, MCL‐1, was significantly decreased during necroptosis; however, this loss was significantly repressed in RIPK3 deleted MEFs, indicating that necroptosis has an influence on mitochondrial death regulatory proteins and the resulting mitochondrial dysfunction [74]. Beclin‐1, another BH3‐domain protein, was reported to be a negative regulator of necroptosis [131, 132]. Beclin‐1 suppressed MLKL oligomerization by interacting with phosphorylated MLKL, followed by its incorporation into the necrosome complex [131]. The inactivation of the *BECLIN‐1* gene sensitized HT‐29, TC‐1, L929, and Molm‐13 cells, to TNF‐α‐induced necroptosis, by facilitating the pore‐formation capacity of oligomerized MLKL [131]. However, the protein, BAD, increased the susceptibility of breast cancer cells to docetaxel‐induced necroptosis [133]. The MLKL inhibitor, necrosulfonaminde [134], significantly inhibited docetaxel‐mediated necroptosis in MDA‐MB‐231 breast cancer cells transfected with BAD, validating the role of BAD in the modulation of necroptosis [133].

In summary, mitochondria play a contextual role in the regulation and induction of necroptosis, mainly through mechanisms that involve ROS generation. Mitochondrial‐mediated necroptosis involves MPT, BCL‐2‐interacting proteins, the RIPK1–RIPK3–MLKL–PGAM5–Cyp‐D–MPTP–DRP1 pathway, and RIPK2–RIPK3 interactions. Mitochondrial dynamics, energy metabolism, and ROS accumulation are all critical regulators of mitochondrial dynamics, energy metabolism, and ROS accumulation.

## 4 Mitochondria in Autophagy Regulation

Autophagy is a lysosome‐dependent cellular degradative process that mediates the bulk recycling of cytoplasmic aggregation proteins and defective organelles [36, 37]. Autophagy is a catabolic process that is evolutionarily conserved that involves the sequestration of cytoplasmic content or organelles into double‐membrane vesicles and are biodegraded after being transported to the lysosomes [135, 136, 137]. There are three types of autophagy: (1) macroautophagy (involves the transport of cytoplasmic contents to lysosomes via double‐membrane vesicle formation called autophagosome; (2) microautophagy (direct degradation of cytosolic contents by lysosome through lysosomal membrane invagination), and (3) chaperone‐mediated autophagy (translocation and degradation of targeted proteins in lysosomes chaperone proteins, such as Hsc‐70) [138].

**FIGURE 3:**
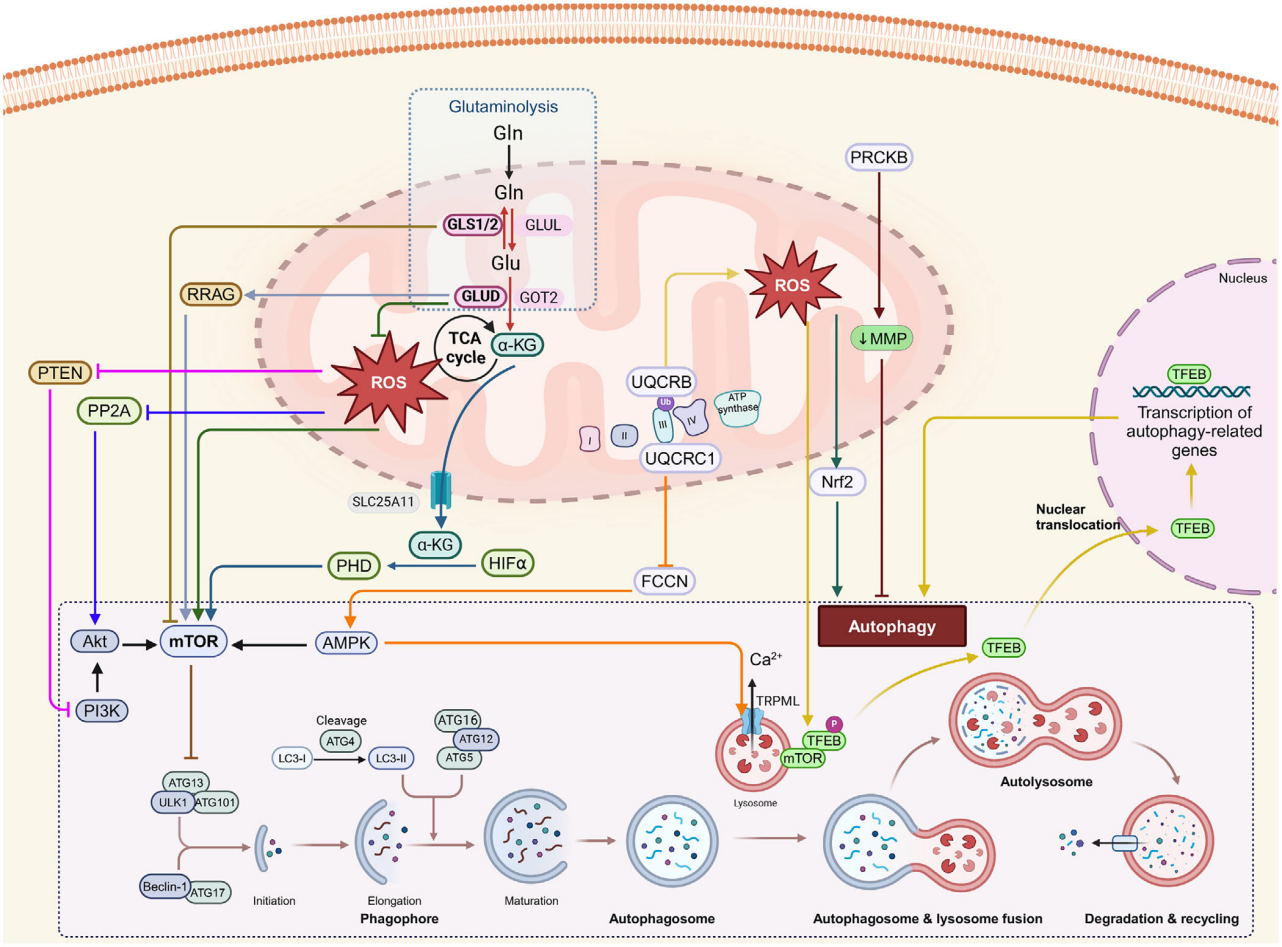
The role of mitochondrial oxidative stress and energetics in autophagy. By inhibiting PI3K–Akt–mTOR‐mediated autophagy, mtROS impedes autophagy through phosphatase and tensin homolog deleted on chromosome ten (PTEN). An increase in mtROS also increases the likelihood of autophagy. Ubiquinol–cytochrome *c* reductase core protein 1 (UQCRB) increases mtROS levels, which results in transient receptor potential cation channel (TRPML) activation and the nuclear translocation of transcription factor EB (TFEB), which facilitates autophagy. The enzymes, glutamate dehydrogenase (GLUD) and α‐ketoglutarate (α‐KG) negatively regulate autophagy. GLUD1 limits mtROS production and activates mammalian target of rapamycin (mTOR). α‐KG inhibits mTOR by activating the enzyme, hypoxia‐inducible factor (HIF) prolyl hydroxylases (PHDs), where glutamine synthetase (GLS), UQCRC1, and protein kinase C beta (PRCKB) positively regulate autophagy. GLS inhibits mTOR and activates autophagy. UQCRC1 upregulates AMPK signaling and induces autophagy by increasing the activity of TRPML. A protein kinase family member, PRKCB, negatively regulates autophagy by causing the loss of the mitochondrial membrane potential (MMP). The figure created with BioRender.com.

**FIGURE 4:**
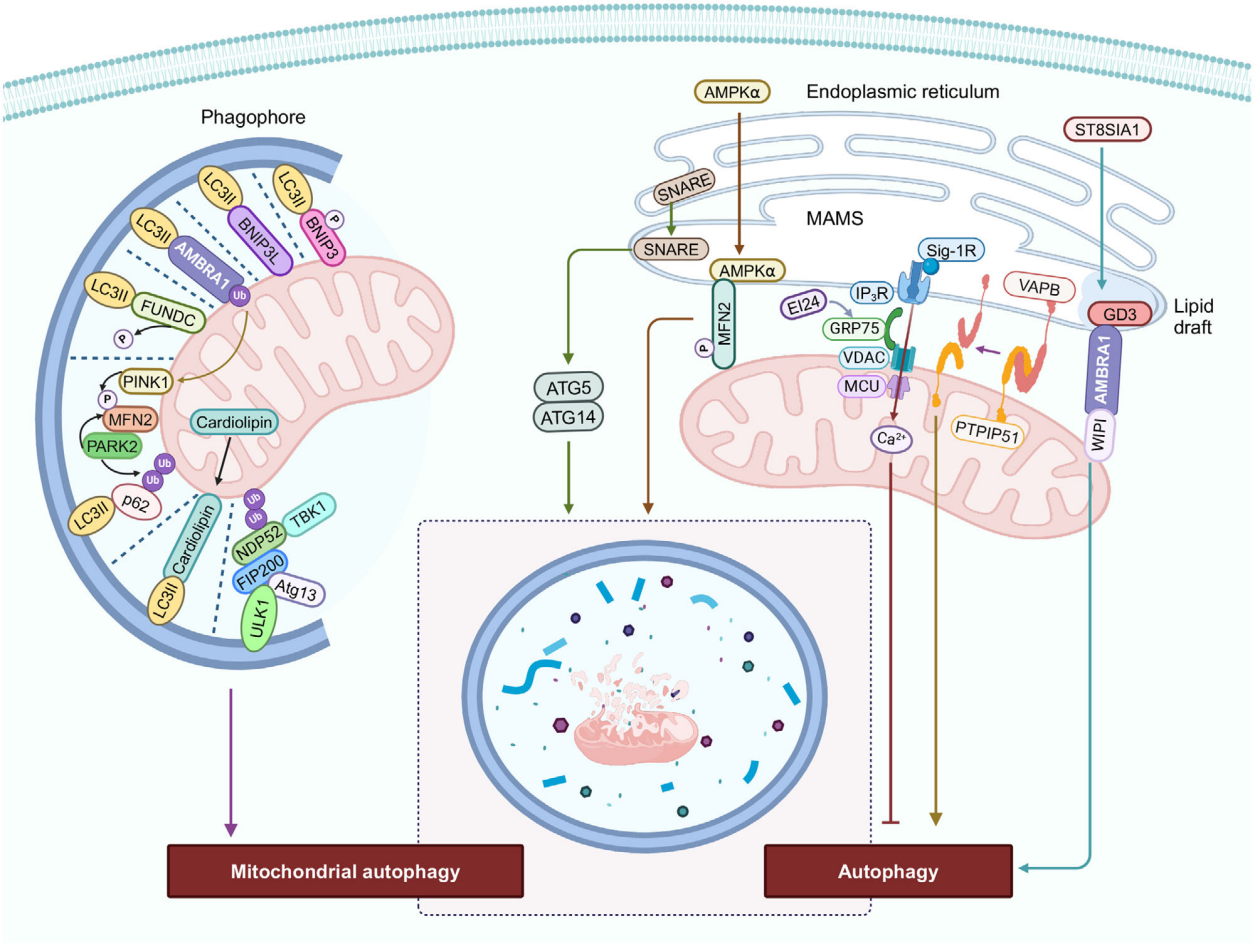
The molecular mechanisms that produce mitophagy and mitochondria‐associated ER membranes (MAMs)‐mediated autophagy. The autophagic recognition of mitochondria occurs through the interaction of the LC3‐interacting region (LIR) of LC3, with several proteins, such as BH3‐only proteins (BNIP3 in its phosphorylated form and BNIP3/Nix), dephosphorylated FUNDC under hypoxic conditions, and the release of activating molecule in beclin‐1‐regulated autophagy (AMBRA1), PTEN‐induced kinase 1–parkin (PINK1–PARK2), and cardiolipin, during mitochondrial hyperpolarization. In PINK1–PARK2‐mediated autophagy, PINK1 is present on the OMM, and it recruits PARK2 by phosphorylating the protein, mitofusin 2 (MFN2). The activation of PARK2 ubiquitinates the protein, p62, which interacts with LC3, producing phagophore formation and ultimately, biodegradation by autophagy. Autophagy receptors, such as nuclear dot protein 52 kDa (NDP52) and tank‐binding kinase 1 (TBK1), can induce autophagy by activating and recruiting the ULK1 (ULK1) complex in the absence of LC3. A decrease in the interaction of the ER and mitochondria due to the loss of St8 alpha‐N‐acetyl‐neuraminide alpha‐2,8‐sialyltransferase 1 (VAPB) and protein tyrosine phosphatase interacting protein 51 (PTPIP51) induces autophagy. MAMs‐associated autophagy is positively regulated by the ER chaperone protein, the sigma‐1 receptor (Sig‐1R) and a ganglioside (GD3), after interaction with AMBRA1 and the WD‐repeat protein, interacting with phosphoInositides (WIPI), the soluble N‐ethylmaleimide‐sensitive factor (NSF) attachment protein receptors (SNARE) protein, and AMPK, upon interaction with phosphorylated MFN2. In contrast, IP3 receptor‐mediated Ca^2+^ negatively regulates MAMs‐associated autophagy. The figure created with BioRender.com.

The inhibition of the protein, mammalian target of rapamycin complex 1 (mTORC1) and the activation of energy sensor AMP‐activated protein kinase (AMPK), induces autophagy by activating a preinitiation complex, composed of Atg1/unc‐51‐like autophagy activating kinase 1 or 2 (ULK1/2), ATG13, and RB1‐inducible coiled‐coil 1 (RB1CC1/FIP200) [139, 140, 141, 142, 143]. The autophagic process consists of five main processes: initiation, elongation of the autophagosome membrane, maturation of the autophagosomes, fusion of the autophagosomes with the lysosomes and degradation [144, 145]. Following the induction macroautophagy, the nucleation of the preautophagosomal membrane, known as the phagophore, occurs at multiple sites, known as phagophore assembly sites [146, 147, 148, 149, 150]. The preinitiation complex recruits and activates an initiation complex consisting of beclin 1, Vps34 (a class III PI3K, phosphatidylinositol kinase), and Vps15 (a serine/threonine protein kinase), which leads to the biosynthesis of the lipid, phosphatidylinositol 3‐phosphate (PI3P) [151]. PI3P is involved in phagophore elongation, which recruits the Autophagy related 7 (ATG7) proteins to the phagophore [152]. There are two conjugation pathways that interact to elongate the phagophore and promote the autophagosomes closure. In the ATG5–ATG12 pathway, ATG7 is a E1 ubiquitin activating enzyme that transfers ATG12 to ATG10, to facilitate the covalent bonding of ATG12 to ATG5 [153]. Conjugated ATG5–ATG12 complexes with ATG16L dimers to stabilize the phagophore [154]. In the LC3 processing pathway, ATG4 cleaves LC3 to generate LC3B‐I, which is conjugated to ATG7 [155]. Activated LC3B‐I is transferred to the E2‐like carrier protein, ATG3, that facilitates LC3B‐I conjugation to phosphatidylethanolamine (PE), to form an LC3‐PE conjugate [139]. This lipidated/processed LC3B‐II located on the internal and external autophagosome membrane plays an important role in membrane fusion and in targeting biosubstrates for degradation [155]. The autophagy substrate/cargo receptor, p62/SQSTM1, interacts with LC3B, to transfer cargo to autophagosomes for degradation [156]. Subsequently, the fusion of autophagosomes with lysosomes forms autolysosomes [139]. This fusion is mediated by RAB7 and the interaction of soluble N‐ethylmaleimide‐sensitive factor attachment protein receptors (SNARE) proteins, such as syntaxin 17, vesicle‐associated membrane protein 8 (VAMP8), and the SNARE‐binding protein synaptosomal‐associated protein of 29 kDa (SNAP29) on the lysosomes [157]. The lysosomal‐associated membrane proteins (LAMPs), LAMP1 and LAMP2, are also critical for fusion events [158].The role of mitochondria in regulating autophagy is summarized in Figures 3 and 4.

### 4.1 Mitochondrial Oxidative Stress and Autophagy

Numerous studies have shown that ROS regulates autophagy and that mitochondria are the primary source of autophagy regulation [159, 160, 161, 162]. The attenuation of autophagy by the ROS scavengers/antioxidants, such as NAC, validates the role of mtROS in the regulation of autophagy [163, 164]. For example, deoxypodophyllotoxin‐induced apoptosis increased mtROS production in PC‐3 cells, which further activated extracellular signal‐regulated kinases/mitogen‐activated protein kinases (MAPK) signaling and induced protective autophagy [165]. Similarly, the loss of solute carrier family 4 member 11 (SLC4A11), a NH3‐sensitive transporter present in the plasma membrane and mitochondria of the corneal epithelium [166], increasedde mtROS, which disrupts the transcription factor EB (TFEB) signaling pathway, contributing to lysosomal dysfunction and impaired autophagy in the corneal endothelium [167]. Furthermore, mtROS produced by the overexpression of ubiquinol cytochrome *c* reductase binding protein (UQCRB) facilitated autophagic flux by lysosomal biogenesis through activation of the transient receptor potential cation channel, mucolipin subfamily 1 (TRPML1) Ca^2+^ channels and TFEB nuclear translocation and protects against HCT116 colorectal cancer [168]. In contrast, the inhibition of mtROS generation by the UQCRB inhibitor, A1938 [169] decreased autophagy in UQCRB‐overexpressing HEK293 cells [168]. Honokiol‐induced cytoprotective autophagy in PC‐3 cells was partly dependent on mitochondria‐derived ROS accumulation, as indicated by the partial suppression of the LC3BII protein level, after incubation with NAC [170]. NAC inhibited recombinant human arginase (rhArg)‐induced autophagy in A375 human melanoma cells, indicating that rhArg‐induced autophagy was mediated by ROS [171]. Another study reported that mtROS generation occurs upstream of autophagy and this produced autophagy in prostate cancer cells [172]. BIX‐01294 (a histone methyltransferase inhibitor [173]) induced ROS‐dependent autophagy in breast cancer cells, by increasing the levels of mitochondrial superoxide and mitochondrial and cytosolic H_2_O_2_ levels [163]. Furthermore, mtDNA‐less human pancreatic ρ0P29 cancer cells that generated minimal ROS levels after incubation with SSHE, an ethanol extract of Syussai ginger, were resistant to autophagy, indicating that SSHE produced mtROS‐mediated autophagy in human pancreatic cells [164]. The mtROS produced by damaged mitochondria has been shown to impair lysosomal function and produce aberrant autophagic flux in macrophages [174]. Interestingly, the increase in the level of the decidual protein, C10ORF10/DEPP, induced by progesterone, a transcriptional target of Forkhead box O3 (FOXO3) that is present in mitochondria [175], mediates ROS accumulation and regulates FOXO3‐induced autophagy in human neuroblastoma cells [176]. Similarly, the attenuation of autophagy by ROS suppressors/antioxidants, such NAC, diphenyleneiodonium chloride (DPI), and mitochondrial superoxide dismutase 2 (SOD2), are similar to manganese (III) tetrakis (4‐benzoic acid) porphyrin (MnTBAP; ROS scavenger) [177], validates the role of mtROS in the regulation of autophagy [163, 164, 176]. In stress conditions, such the exposure of human retinal pigment epithelial cells (ARPE‐19) to ethanol, mtROS levels are increased and mitochondrial fission activation occurs, activating the autophagy pathway [178]. In vitro, metformin increases mtROS in CD8+ tumor‐infiltrating T cells (CD8TILs), which in turn activate nuclear factor erythroid 2‐related factor 2 (Nrf2), resulting in mTORC1 activation, phosphorylation of p62 and the induction of autophagy [179]. The inhibition of autophagy in prostate cancer (PC‐3 cells), human retinal pigment epithelium (ARPE‐19 cells), and CD8TILs, after incubation with mitochondria‐targeted antioxidant drugs, such as mitoquinone (MitoQ), mitoTEMPO, or the mitochondrial SOD2 mimic, manganese (III) tetrakis (4‐benzoic acid) porphyrin (MnTBAP), confirms the role of mtROS in the regulation of autophagic response [165, 178, 179].

In contrast, another study suggested that mtROS inhibits phosphatase and tensin homolog deleted on chromosome ten (PTEN) enzyme, a primary downstream effector of the PI3K–Akt–mTOR signaling pathway, which catalyzes the biodegradation of PIP3, thereby inhibiting PI3K signaling [180]. Furthermore, high levels of mtROS have been reported to activate Akt by downregulating phosphoprotein phosphatase 2A, which plays a crucial role in AKT inactivation [181] and positively regulates the PI3K–Akt–mTOR signaling pathway, thus inhibiting autophagy signaling [182].

### 4.2 Mitochondrial Energetics and Autophagy

Cancer cells require a significant supply of nutrients, particularly glucose and glutamine, to synthesize macromolecules and replenish the tricarboxylic acid (TCA) cycle, to meet the energy required for their proliferation [183]. It has been reported that amino acids, such as glutamine, inhibit macroautophagy before the fusion of the autophagosomes and lysosomes [184] and inhibit the starvation of glutamine induced prosurvival autophagy in colorectal carcinoma cells [185]. Glutamine deprivation has been reported to translocate and activate TFEB, producing micropinocytosis‐induced autophagy in pancreatic ductal adenocarcinoma cells [186]. During amino acid deprivation, autophagy induces the reactivation of mTORC1, a major regulator of cell growth and autophagy [187, 188]. The activation of mTORC1 signaling produces an addiction to glutamine in cancer cells [189]. Amino acid deprivation depletes α‐ketoglutarate (α‐KG), which further inactivates HIF prolyl hydroxylases (PHDs), preventing mTORC1 activation and inducing autophagy [190]. In cells with high levels of glutaminolysis, cytosolic α‐KG is transported from mitochondria to the cytosol by mitochondrial carrier protein, solute carrier family 25 member 11 (SLC25A11 [188]), activates PHDs, which further increases amino acid‐dependent‐mTORC1 activation in a HIF‐1α‐independent manner, inhibiting autophagy [188, 190, 191]. Furthermore, activating transcription factor 4 is activated upon glutaminolysis inhibition, increasing the levels of DNA damage inducible transcript 4, which inhibits mTOR and increases the likelihood of prosurvival autophagy [192]. Similarly, the enzyme, GLUD, which mediates the catalysis of glutamate to α‐KG, modulates autophagy by limiting the production of ROS and activating mTORC1, based on autophagy initiation after GLUD1 knockdown, which was characterized by an increase in ROS levels and a decrease in mTORC1 activation [193]. GLUD also inhibited autophagy by the GTP‐mediated activation of RAS‐related GTPase and the subsequent activation of mTORC1 [193, 194]. Under conditions of amino acid deprivation, FOXO3 increased the expression of glutamine synthetase, which inhibited mTOR and increased autophagy in human colon cancer DLD1 cells [195]. The inactivation of glutamine synthetase decreased FOXO‐mediated LC3II formation and decreased autophagy, indicating that glutamine synthetase, which catalyzes the conversion of glutamate and ammonia to glutamine, plays a role in the regulation of autophagy [195]. Chronic respiratory chain deficiency due to the knockdown of ubiquinol‐cytochrome *c* reductase core protein 1 (UQCRC1), a subunit of respiratory chain complex III [196], produced an increase in folliculin expression, which downregulated AMPK signaling and decreased phosphatidylinositol 3,5‐bisphosphate (PI(3,5) P2) levels [197]. The knockdown of UQCRC1 further decreased mucolipin TRP cation channel 1 activity, producing the accumulation of Ca2+ in the lysosomes, increasing lysosomal volume and the accumulation of autophagosomes, which inhibited autophagy [197]. PRKCB, a member of the protein kinase C (PRKC) family, inhibited autophagy by increasing the loss of ΔΨM and negatively regulating mitochondrial energy [198]. This finding was validated based on data indicating that PRKCB‐depleted MEFs had increased autophagy with restored mitochondrial bioenergetics, based on an increase in ΔΨM [198]. These data indicate autophagy can be regulated by the bioenergetics state of mitochondria.

### 4.3 Mitochondria Degradation by Autophagy

The selective degradation of mitochondria by autophagy is known as mitophagy [199, 200]. Mitophagy is critical for the turnover of mitochondria and the removal of unhealthy and damaged mitochondria [199]. Mitophagy is regulated by proteins that are involved in the maintenance of mitochondrial integrity, morphology, and ubiquitination [201]. Mitochondria undergo fission to separate damaged mitochondria from the healthy mitochondria, which are subsequently removed by mitophagy [202]. In damaged mitochondria, PINK1 is accumulated on the OMM due to impaired intermembrane transport to IMM [40]. PINK1, upon phosphorylation at serine 228 and 402, recruits the enzyme, cytosolic E3 ubiquitin ligase, and PARK2, to the OMM [203]. The protein, autophagy‐regulating protease 4 (ATG4), is also involved in PINK1/Parkin‐mediated mitophagy. Indeed, ATG4 interacts with ATG9A and increases the interaction between the phagophore and ER, promoting mitophagy independent of LC3/the γ‐aminobutyric acid receptor‐associated proteins (GABARAP) lipidation system [204]. Similarly, autophagy receptors, such as Nuclear dot protein 52 kDa (NDP52) (also known as calcium‐binding and coiled‐coil domain 2) and the multifunctional autophagy kinase, TANK‐binding kinase 1 (TBK1), activate mitophagy downstream or independent of the PINK1/Parkin axis, by recruiting and activating the ULK1 complex without binding to LC3 proteins [205].

BH3‐only proteins, such as the Bcl‐2 interacting protein 3 (BNIP3) and BNIP3L (BNIP3‐like)/Nix, also mediate mitophagy. The phosphorylation of Ser34 and Ser35 juxtaposed at the LC3 interacting region (LIR) domain of BNIP3L, increases its interaction with ATG8 family proteins, such as LC3/GABARAP and increases mitophagy in HeLa cells [206]. The accumulation of BNIP3L on the OMM, to a certain level, induces the loss of ΔΨM, producing dysfunctional mitochondria, which activates mitophagy [207, 208, 209, 210, 211, 212]. Furthermore, BNIP3 and BNIPL inhibit mTOR activity by binding with Ras homolog enriched in brain (Rheb) proteins, which facilitate mitophagy [213]. Similarly, under hypoxic conditions, the BH3‐domains of BNIP3 and BNIP3L release Beclin‐1 by competing with Beclin1–Bcl‐2 and Beclin1–Bcl‐Xl complexes, thus inducing the autophagy of mitochondria to prevent ROS and cell death [214, 215, 216]. In contrast, hypoxic‐colon carcinoma‐induced mitophagy was reported to be independent of BNIP3 and BNIP3L and was directed by the activation of the AMPK–ATG5 axis [217]. Furthermore, another protein involved in mitophagy is activating molecule in activating molecule in beclin‐1‐regulated autophagy (AMBRA1). Under basal condition, AMBRA1 is located in the mitochondria and its proautophagic activity is inhibited by Bcl‐2 [218]. Mitophagy induction after mitochondrial depolarization leads to the binding of AMBRA1 to LC3 via its LIR motif, producing Parkin‐mediated autophagy [219]. Intriguingly, high levels of AMBRA1 can induce mitophagy, even in cells lacking Parkin or PINK1, indicating that AMBRA1 mediates mitophagy independent of Parkin, PINK1, or p62‐recruitment [219]. Under hypoxic conditions, dephosphorylated FUN14 domain containing 1 (FUNDC1), a OMM protein, binds with LC3B via its LIR and activates mitophagy [220]. The absence of mitochondria engulfment by autophagosomes and intact mitochondrial integrity with no biodegradation of mitochondrial proteins in FUNDC1‐silenced cells, indicated that FUNDC1 is involved in mitophagy under hypoxic conditions [220]. The knockdown of the gene for the mitochondrial fission protein DNM1L/DRP1, prevented carbonyl cyanide‐p‐trifluoromethoxyphenylhydrazone‐ and selenite‐induced mitophagy [221]. However, the knockdown of the gene for the mitochondrial fusion protein, OPA1, increased mitophagy, suggesting that FUNDC1 interacts with OPA1 and DNM1L to induce mitochondrial fission and mitophagy [221].

### 4.4 Mitochondrial Membrane Contribution to Autophagy

Mitochondria are one of the organelles, besides the ER and Golgi bodies, that are source of membrane and lipids for the formation, expansion, and fusion of autophagosomes [222]. The membrane contact sites (MSCs) for autophagosome biogenesis include ER–phagophore MSCs, ER–mitochondria MSCs, mitochondria‐associated ER membranes (MAMs), ER–plasma membrane MSCs, ER–Golgi intermediate compartments (ERGIC), and plasma‐membrane‐derived vesicles [205, 223–225]. During nutrient deprivation, mitochondria are involved in autophagosome biogenesis, as they transfer lipids from its outer membrane to newly formed autophagosomes, following the recruitment of the early and late autophagosome markers, ATG5 and LC3, on mitochondria [222]. The ER‐resident SNARE protein, syntaxin 17, translocates to MAMS after nutrient deprivation and recruits the preautophagosome/autophagosome marker, ATG14 and the autophagosome‐formation marker, ATG5, to the MAMs, to produce autophagosomes [226]. Furthermore, the formation of MAMs occurs by the tethering of the ER protein, VAMP‐associated protein B (VAPB), to the mitochondrial protein, protein tyrosine phosphatase‐interacting protein 51 (PTPIP51), followed by ER Ca^2+^ delivery to mitochondria [227, 228, 229, 230]. The inactivation of the gene for VAPB and PTPIP51 decreases the mitochondria contact sites and increases autophagosome formation, inducing autophagy [227]. The overexpression of VAPB and PTPIP51 increases the ER–mitochondria contact sites and inhibits rapamycin‐ and torin 1‐induced autophagosome formation, which impairs autophagy [227]. Autophagy was restored by decreasing the IP3‐receptor‐mediated Ca^2+^ supply to mitochondria in cells overexpressing VAPB and PTPIP51 [227], indicating that autophagy is activated when Ca^2+^ transport from MAM‐located IP3 receptors to mitochondria is disrupted [227, 231–233]. Another ER protein involved in autophagy regulation is etoposide‐induced protein 2.4 (EI24). EI24 interacts with glucose‐regulated protein 75, an OMM chaperone protein, that mediates MAM integrity by forming a complex with VDAC1 and the inositol 1,4,5‐trisphosphate receptor (IP_3_R) [234, 235]. A deficiency of EI24 affects Ca^2+^ transfer from the ER to mitochondria and inhibits autophagy [234]. Similarly, the sigma‐1 receptor (Sig‐1R), an ER chaperone protein located primarily at the MAM, also regulated Ca^2+^ signaling between ER and mitochondria and its ablation or suppression impaired the assembly and clearance of autophagosome [236, 237]. Interestingly, under energy stress, cytosolic AMPKα1 was translocated to MAMs tethered to MFN2, where it interacted with and phosphorylated MFN2 [238]. This, in turn, increased MAMs levels, inducing autophagy. Furthermore, MFN2‐depleted cells had a significant decrease in autophagic capacity and defective MAM production, indicating the importance of the AMPK–MFN2 axis in the induction of autophagy [239]. Lipid rafts in MAMs containing ganglioside GD3, also known as the brick of lipid rafts [240], interacts with AMBRA1 and WD repeat domain phosphoinositide‐interacting protein 1 (WIPI1), to initiate autophagy [241]. Furthermore, the depletion of the enzyme, ST8 alpha‐N‐acetyl‐neuraminide alpha‐2,8‐sialyltransferase 1 (ST8SIA1), which is involved in ganglioside production, decreased ER–mitochondria interaction and impaired the nucleation of autophagosomes [241, 242]. In conclusion, MAM plays a role in autophagosome biogenesis and disruption of MAMs or ER–mitochondria contact inhibits autophagy.

Mitochondria regulate autophagy via diverse mechanisms, including ROS signaling, energy metabolism, and mitochondrial mitophagy among others. For example, mitochondria‐generated ROS can induce or inhibit autophagy, whereas mitochondrial bioenergetics modulate autophagic responses under stress or nutrient‐depleted conditions. To maintain cellular homeostasis, mitochondria are specifically targeted for degradation by mitophagy. Furthermore, mitochondria‐associated membranes (MAMs) facilitate autophagosome formation by providing structural components and mediating Ca^2^⁺ transfer from mitochondria to the ER. Mitochondria play a key role in autophagy regulation by integrating metabolic and signaling cues. Thus, mitochondria regulate autophagy by integrating metabolic and signaling cues to control cellular health.

## 5 Mitochondria in Ferroptosis Regulation

Ferroptosis is a nonapoptotic, iron‐dependent‐regulated cell death, characterized by excessive lipid peroxidation due to inhibition of system Xc−, a cystine/glutamate antiporter or glutathione peroxidase (GPX4), an antioxidant lipid repair enzyme [243]. System Xc− consists of two transmembrane components, SLC7A11 and SLC3A2, which regulate the intake of extracellular cystine into the cell, in exchange for glutamate [244, 245]. Cystine then undergoes reduction to cysteine, which serves as a precursor for the biosynthesis of glutathione (GSH), a γ‐l‐glutamyl‐l‐cysteinyl‐glycine tripeptide [246]. GPX4 oxidizes GSH to GSSH, which decreases phospholipid (PL) hydroperoxides and oxidized lipoproteins within the cellular membranes [247]. Alterations in the GSH–GPX4 axis, such as the inhibition of system Xc−, decreases the cellular levels of cysteine, decreases the biosynthesis of GSH (an important cofactor for GPX4) by direct inhibition of GPX4, increases lipid peroxidation, contributing to ferroptotic cell death [248]. Another protein that regulates ferroptosis is GTP cyclohydrolase‐1 (GCH1). GCH1 expression increases the production of tetrahydrobiopterin (BH4) and dihydrobiopterin (BH2), which facilitates lipid remodeling by preventing the depletion of PLs that have two polyunsaturated fatty acyl tails and inhibits ferroptosis without affecting other ferroptosis regulators [249]. Ferroptosis is also modulated by the protein, ferroptosis suppressor protein 1 (FSP1), which mediates the NADPH‐dependent reduction of ubiquinone (coenzyme Q10 or CoQ10), a lipophilic antioxidant that eliminates peroxyl radicals on plasma membranes, inhibiting lipid peroxidation and subsequently, ferroptosis [250, 251]. Among the membrane lipids, polyunsaturated fatty acids (PUFAs) are the most susceptible to peroxidation [252, 253]. PUFAs are ligated with coenzyme A (CoA) and re‐esterified into PLs by acyl‐CoA synthetase long‐chain family member 4 and lysophosphatidylcholine acyltransferase 3 enzymes, respectively [254, 255]. The PLs then undergo peroxidation by: (1) enzymatic catalysis by certain cytochrome P450 oxidoreductases or lipoxygenases or (2) nonenzymatic catalysis mediated by Fe^2+^ via the Fenton reaction [256]. This produces reactive hydroxyl and peroxyl radicals that cause the peroxidation of PUFA–PLs, thereby increasing the likelihood of ferroptosis [256].

Overall, the disruption of lipophilic antioxidant system, such as GSH–GPX4, GCH1–BH4/BH2, and the FSP1–CoQ–NADPH axis, produces the lethal accumulation of lipid peroxides that compromise biological lipid bilayer membrane fluidity, permeability, and overall structure, inducing cell death [248, 257].

**FIGURE 5:**
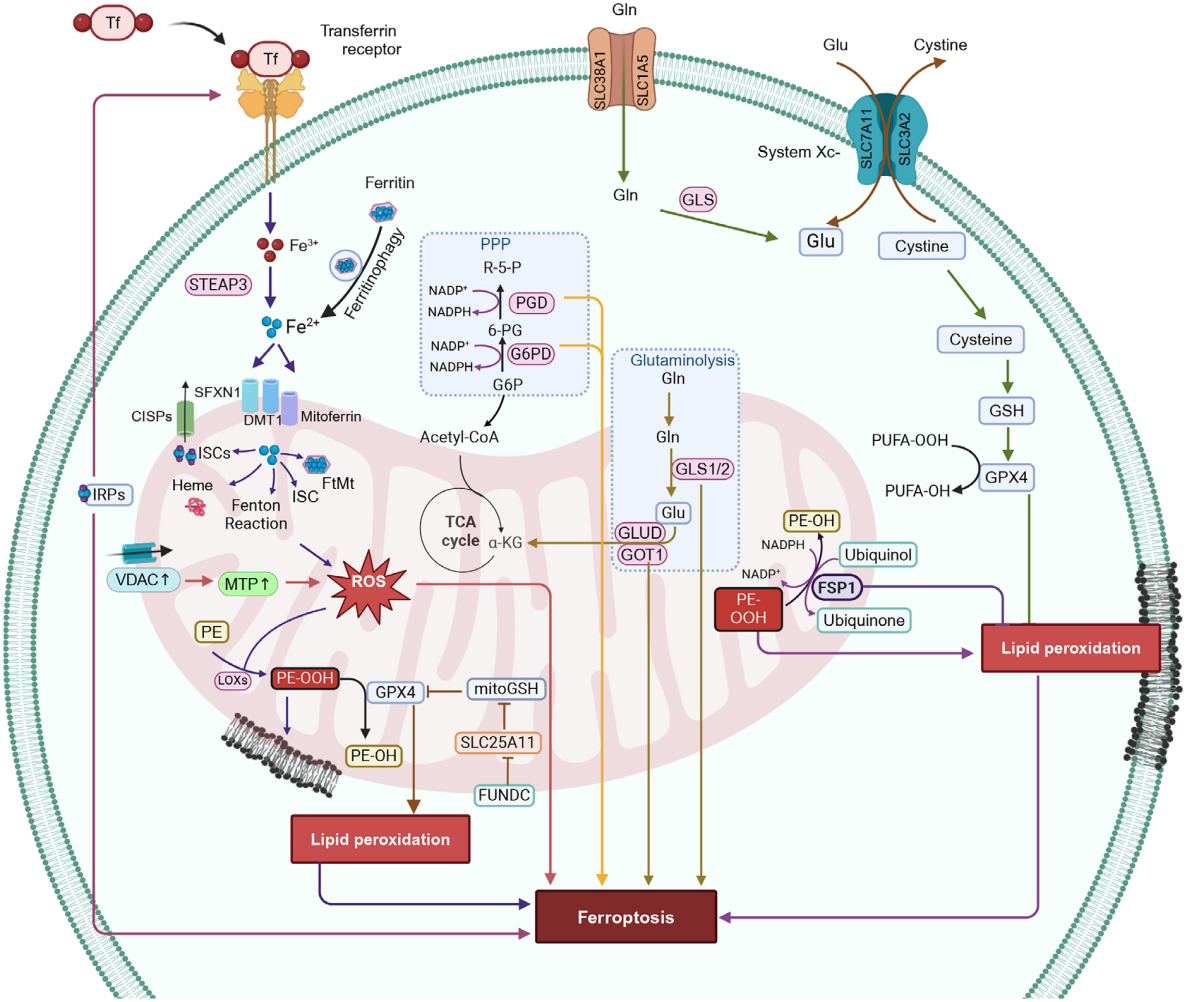
The role of mitochondria in ferroptosis. Opening of voltage‐dependent anion channels (VDAC) increases the mitochondrial transmembrane potential and leads to mitochondrial hyperpolarization, which increases the levels of mtROS. Increased cellular Fe^2+^ levels by the Fenton reaction, ferritinophagy, mitochondrial ferritin, iron–sulfur cluster or upregulation of iron regulatory protein 2 (IRP‐2), also contribute to mtROS production. MtROS increases the likelihood of ferroptosis by increasing lipid peroxidation. The enzymes involved in the pentose phosphate pathway, such as glucose‐6‐phosphate dehydrogenase (G6PD) and phosphoglycerate dehydrogenase (PGD) and enzymes involved in glutaminolysis, such as glutaminase (GLS) and glutamic‐oxaloacetic transaminase 1 (GOT1), are positive regulators of ferroptosis. Ferroptosis suppressor protein 1 (FSP1) suppresses lipid peroxidation and inhibits ferroptosis. The figure created with BioRender.com.

Mitochondria undergo ultrastructural changes upon induction of ferroptosis. The incubation of human foreskin fibroblasts, BJeLR cells, that express hTERT, LT, ST, and an oncogenic *HRAS* allele, to the compound, erastin (an initiator of ferroptotic cell death [258]) resulted in smaller mitochondria with higher membrane density and a decreased number of cristae [258]. A similar mitochondrial phenotype occurred in *GPX4*‐deleted cells Pfa1 cells incubated with the compound, buthionine sulfoximine [259], an irreversible inhibitor of glutamylcysteine synthetase [260], which is involved in the synthesis of GSH [261]. These cells had swollen mitochondria with lamellar and tubular structures and diminished cristae [259, 262]. Similarly, the incubation of Pfa1 cells with the ferroptotic inducer, RSL3, an inhibitor of GPX4, produced a time‐dependent rupture of the OMM [259]. Furthermore, the mitochondria in ferroptotic cells can be categorized into four groups, based on the magnitude of mitochondrial fragmentation and subcellular location: (i) evenly distributed, elongated mitochondria; (ii) uniformly distributed, fragmented mitochondria; (iii) fragmented mitochondria, primarily distributed around the nucleus, and (iv) small spherical mitochondria around the nucleus [263, 264, 265, 266]. Overall, the mitochondrial changes after the induction of ferroptosis include mitochondrial fragmentation, increased mitochondrial membranes density, OMM rupture, and diminished mitochondrial cristae. The role of mitochondria in regulating ferroptosis is summarized in Figure 5.

### 5.1 Mitochondrial Lipids Affect Ferroptosis

Cardiolipins (CLs), a unique PL found exclusively in the inner mitochondrial membrane (IMM) and other PLs, such as phosphatidylcholine (PC) and PE, undergo oxidative reactions in the kidneys of Gpx4^−/−^ mice and in HT‐1080 cells incubated with FINO2, an inducer of ferroptosis [259, 267]. The knockdown of *SMPD1* and *TAZ* (tafazzin) genes, which mediate CLs remodeling [268], exacerbated acetaminophen‐induced mitochondrial dysfunction and ferroptosis in hepatocytes [262]. The oxidized PLs and CLs primarily accumulated in the IMM of ferroptotic cells [259]. According to Gao et al. [269], lipid peroxides are co‐localized in the mitochondria at early time points and in the plasma membrane at later time points. Similarly, doxorubicin (DOX)‐induced ferroptosis is characterized by increased lipid peroxidation in mitochondria but not in the cytoplasm of cardiomyocytes [270, 271]. MitoTEMPO, an antioxidant that targets mitochondria [272] but not TEMPO, a nonmitochondria targeted antioxidant, abolished DOX‐induced lipid peroxidation and subsequent ferroptosis, indicating that the oxidative damage induced by mitochondria is a major mechanism of ferroptosis induction [271]. The efficacy of the mitochondria‐targeted nitroxide antioxidant, XJB‐5‐131, to rescue FINO2 (endoperoxide‐containing 1,2‐dioxolane)‐induced ferroptotic death of HT‐1080 cells, was 39‐fold greater than that of the cytosolic targeted antioxidant, JP4‐039 [273], highlighting the critical role of mitochondrial lipid peroxidation in ferroptosis [267]. However, only a few studies have shown the accumulation of lipid peroxides to be extra‐mitochondrially, and it occurs primarily in the ER [253].

### 5.2 Mitochondrial Dynamics in Ferroptosis

Mitochondria establish an electrochemical proton gradient across the mitochondrial membrane, commonly known as MMP, by utilizing oxidizable substrates, which produces ATP [274]. Several ferroptosis inducers, such as erastin, an amino acid‐free medium, glutamate, and cysteine deprivation, resulted in MMP hyperpolarization [269]. The incubation of human fibrosarcoma HT1080 cells with the mitochondrial OXPHOS uncoupler, carbonyl cyanide m‐chlorophenyl hydrazone [275], impaired MMP, depolarized mitochondria and prevented the accumulation of lipid peroxides, due to cysteine deprivation and ferroptosis [269]. Erastin binds to the VDACs, VDAC2, and VDAC3 [276], whereas RSL3 produced a conformational change in VDAC2 [277] by inducing carbonylation [278]. VDAC facilitated the flux of ions and metabolites across the OMM [279]. The binding of erastin and RSL3 prevented the tubulin‐dependent closure of VDAC, thus producing mitochondrial hyperpolarization [278]. The knockdown of the VDAC2/3 genes prevented erastin‐induced ferroptosis [280]. In contrast, acetaminophen‐induced ferroptosis in hepatocytes, as well as erastin‐ or RSL3‐induced ferroptosis, in MEFs, HT‐22, HepG2, and HA22T/VGH cancer cells, resulted in the loss of the MMP [262, 263, 265, 281]. Ferroptosis is also regulated by the mitochondrial outer membrane protein, FUN14 domain‐containing 2 (FUNDC2). FUNDC2 interacts with and destabilizes the mitochondrial GSH transporter, SLC25A11, decreasing mitoGSH levels, thus, contributing to DOX‐induced ferroptosis [282]. The ablation of the FUNDC2 gene prevented DOX‐induced cardiomyopathy by ferroptosis and the knockdown of the SLC25A11 gene in FUNDC2‐KO cells significantly decreased mitoGSH levels and augmented erastin‐mediated ferroptosis [282].

### 5.3 Mitochondrial Energetics in Ferroptosis

The major function of mitochondria is to produce ATP by the process of OXPHOS that involves electron transport and chemiosmosis [283]. During this process, the transfer of electrons during the production of ATP leads to the generation of ROS, such as hydrogen peroxide (H_2_O_2_), hypochlorous acid (HOCl), hydroxyl radical (HO^•^), alkoxyl radical (RO^•^), superoxide anion (O_2_^•−^), peroxyl radical (RO_2_^•^), hydroperoxyl radical (HO_2_^•^), and singlet oxygen (^1^O_2_) [284]. The enzyme, NADPH oxidase (NOX), and the family of superoxide‐producing enzymes, such as NOX1‐5 and DUOX1‐2, have been shown to produce lethal levels of ROS that activated erastin‐mediated ferroptosis [280]. The erastin‐induced ferroptotic cell death in human non‐small‐cell lung cancer Calu‐1 cells was inhibited by DPI (a NOX inhibitor [285]), GKT137831 (a NOX1/4‐specific inhibitor [286]), and 6‐aminonicotinaminde (6‐AN, a NADPH‐producing pentose phosphate pathway (PPP) inhibitor [287]), suggesting that ferroptosis is induced by NADP‐dependent ROS production [280]. Similarly, the knockdown of the genes for the PPP enzymes, glucose‐6‐phosphate dehydrogenase and phosphoglycerate dehydrogenase, which are involved in NADPH production [288], rescued the erastin‐induced ferroptosis in Calu‐1 cells [280]. In contrast, erastin‐induced ferroptosis in HT‐1080 fibrosarcoma cells was only moderately inhibited by the NOX inhibitors, DPI and GKT137831, and 6‐AN [258]. This finding suggested that in addition to the PPP/NOX pathway, other pathways can mediate ferroptosis after the initial inhibition of system X_c_^−^ [280]. High levels of mtROS were reported in ferroptotic death induced by glutamate in HT‐22 mouse hippocampal neurons cells, acetaminophen in Hepa1‐6 murine hepatoma cells, and RSL3‐challenged MEF and HT‐22 cells [262, 265, 289].

Studies indicate that ferroptosis is dependent on cellular metabolism [290]. Numerous studies have reported that mitochondrial dysfunction causes a switch from aerobic respiration to glycolysis for ATP production in cancer cells, known as the Warburg effect [291]. However, mitochondrial failure and increased glycolysis in ferroptosis appear to be unrelated [258, 292]. Erastin‐induced ferroptosis increased ATP synthesis and decreased the rate of glycolysis [258, 292]. Erastin‐induced ferroptotic cell death involved the opening of VDAC in HepG2 and Huh7 human hepatomacarcinoma cells [278]. This erastin‐mediated effect permitted the flux of ATP, ADP, Pi, and metabolite exchange across the OMM, thus promoting mitochondrial metabolism, increased MMP, and increased mtROS levels, followed by mitochondrial dysfunction and the ferroptotic death of HepG2 cells [278, 293]. Cells with greater mitochondrial respiration and lower glycolytic flux, such as HepG2 cancer cells, were more vulnerable to RSL3‐induced ferroptosis, highlighting the specific function of mitochondria in RSL3‐induced ferroptosis [281]. Interestingly, mitochondrial‐deficient cells are less vulnerable to ferroptosis inducers, such as erastin and cysteine deprivation, with cysteine‐deprived mitochondrial depleted cells being more resistant to ferroptosis than cells incubated with erastin [269]. Recent studies have shown that the glutaminolysis–TCA cycle, in combination with the ETC, play an essential role in ferroptosis regulation [294, 295]. Glutaminolysis is the process by which cellular glutamine is biotransformed to glutamate, which is biotransformed to α‐KG, to drive the TCA cycle by anaplerosis [296, 297, 298]. Glutamine is a carbon source for the TCA cycle and a nitrogen source for the de novo synthesis of amino acids, nucleotides and hexosamines, which is used by cells that are growing and proliferating [299, 300]. Glutamine absorption occurs via the glutamine importers, SLC1A5/SLC38A1, and glutamine is biotransformation to glutamate by glutaminase and eventually to α‐KG, either by glutamic‐oxaloacetic transaminase 1 (GOT1) or GLUD1‐mediated glutamate deamination [301]. The glutaminolysis regulating genes, SLC38A1 and glutaminase 2 (GSL2), positively regulated ferroptosis [294]. Similarly, miR‐137, a tumor suppressor microRNA in several cancers, such as ovarian cancer, pancreatic cancer [302], inhibited ferroptosis by directly inhibiting SLC1A5 [303]. Furthermore, l‐gamma‐glutamyl‐p‐nitroanilide, an inhibitor of the SLC1A5 (ASCT2) and SLC38A1 (SNAT1) transporters [304], decreased lipid peroxide levels and rescued erastin‐induced ferroptosis in melanoma cells [303]. Furthermore, the knockdown of mitochondrial enzyme glutaminase 2 (GLS2) (catalyzes the biotransformation of glutamine to glutamate) but not GLS1 or pharmacological inhibition of GLS, by compound 968 [305], significantly inhibited ferroptosis [299]. The inhibition of transaminases by amino‐oxyacetate or the inactivation of the gene for of GOT1 but not GLUD1, prevented ferroptosis [295]. Interestingly, the supplementation of α‐KG with downstream TCA cycle metabolites, such as succinate, fumarate, and malate, substituted for glutamine and induced ferroptosis in MEFs and HT1080 human fibrosarcoma cells in the presence of cysteine deprivation of inhibition of system Xc− [269, 295, 303]. Furthermore, inhibitors of ETC components, such as rotenone (mitochondrial complex I inhibitor), diethyl butylmalonate (complex II inhibitor), antimycin (complex III inhibitor), and sodium azide (NaN_3_, complex IV inhibitor), prevented ferroptosis by erastin or cysteine deprivation, suggesting that the ETC mediated the induction of ferroptosis [269]. In contrast, recent studies have shown that energy stress activates AMPK, an enzyme that is an energy crisis sensor in tumor cells, to produce ATP, and this inhibited ferroptosis [290, 306–308]. Furthermore, AMPK phosphorylates and downregulates acetyl‐CoA carboxylase 1 and 2 (ACC1/2), thereby decreasing the biotransformation of acetyl‐CoA to malonyl Co‐A, inhibiting the production of PUFAs, ultimately blocking cysteine deprivation and GPX4 inhibition‐mediated ferroptosis [307]. The inhibition of the protein liver kinase B1 (also known as serine/threonine kinase 11), an upstream kinase that activates AMPK [309], increased the likelihood of ferroptosis in non‐small cell lung cancer cells [308]. Overall, these findings show the importance of mitochondrial metabolism in regulating lipid peroxidation and ferroptosis via metabolic processes, such as the glutaminolysis–TCA cycle–ETC axis.

### 5.4 Mitochondrial Iron Metabolism in Ferroptosis Regulation

Mitochondria play an important role in the metabolism, utilization, and homeostasis of cellular iron [310]. Extracellular iron is internalized into cells by the transferrin receptor (TFR)‐mediated endocytosis of transferrin‐bound iron (Fe^3+^), followed by the six‐transmembrane epithelial antigen of prostate 3 (STEAP3)‐mediated reduction of Fe^3+^ to Fe^2+^, direct absorption of Fe^2+^ through divalent metal transporter 1 (DMT1), zinc transporter 8, or heme import and its biotransformation to iron by heme oxygenase (HO) [310, 311, 312, 313, 314]. The efflux or export of Fe^2+^ is regulated by the transporter, ferroportin 1 (FPN), the only known transporter that exports elemental iron from cells [315]. Typically, cellular iron is either bound to heme or is present in iron–sulfur cluster (ICS) or stored as Fe^3+^ in the iron storage protein, ferritin [316]. Ferritin releases Fe^3+^ upon its biodegradation through proteasome‐mediated or autophagic process, also known as ferritinophagy [317]. Ferroptosis occurs in the presence of dysfunctional iron metabolism. The upregulation of the iron regulatory protein 2 (IRP‐2) increased ferroptosis by producing an increase in cellular iron intake by increasing the expression of TFR‐1 [318]. The immuno‐induced depletion of transferrin in serum or by inactivating the TFR gene, prevented MEFs ferroptotic cell death [295]. Furthermore, the knockdown of the gene that codes for the protein, IRP‐2 and the incubation of d‐gal‐induced aged PC12 rat adrenal medullary tumor cells with the iron chelators, deferoxamine, ciclopiroxolamine, 2, 2‐bipyridyl, or iron‐free bovine apo‐transferrin, suppressed ferroptotic cell death [295, 318]. Overall, these findings suggested that the iron carrier protein, transferrin, is required for ferroptotic cell death.

Iron is transported into the mitochondrial matrix by the DMT1, siderofexin (SFXN1), or mitoferrin, where it is utilized for the biosynthesis of heme or ISCs or is stored as mitochondrial ferritin [6]. Interestingly, an increase in cellular Fe^2+^ levels via ferritinophagy, increased the expression of SFXN1 (has mitochondrial serine transporter activity [319]) on the mitochondrial membrane [320]. This process increased the entry of cytoplasmic Fe^2+^ to mitochondria, increasing mtROS levels and the induction of ferroptosis, in sepsis‐induced cardiac injury [320]. The incubation of cardiomyocytes with apelin‐13 (an endogenous ligand for the APJ receptor involved in regulating fluid homeostasis, cardiovascular function, and insulin sensitivity [321]) produced excessive ferritinophagy and subsequent SFXN1‐mediated mitochondrial iron accumulation, producing mtROS formation and ferroptosis [322]. In contrast, HO‐1 has been reported to have a dual (protective as well as detrimental) role in the regulation of ferroptosis [323]. The RNA interference silencing of HO‐1 facilitated ferroptosis by erastin in hepatocellular carcinoma cells (HCC) and renal proximal tubular epithelial cells and ferroptosis produced by sorafenib, an inhibitor of system Xc− in HCCs [324, 325]. However, in HT‐1080 fibrosarcoma cells, hemin, an HO‐1 substrate that increases the expression and enzymatic activity of HO‐1, increased erastin‐induced ferroptotic cell death [326]. However, the incubation of HCC with bilirubin or biliverdin (a metabolite produced by the biotransformation of heme to Fe^2+^ [327]) did not retain erastin‐induced ferroptosis [328]. The induction of ferroptotic death by BAY 11–7085, an inhibitor of IκBα phosphorylation that produces NF‐κB inactivation [329], in triple‐negative breast cancer and glioblastoma cells, involved the upregulation of HO‐1, and HO‐1's effect was decreased by the specific HO‐1 inhibitor, zinc protoporphyrin‐9 [330]. Furthermore, HO‐1 upregulation was the major mechanism involved in DOX‐induced ferroptosis in cardiomyocytes [271]. In these studies, the biodegradation of heme by HO‐1 increased free iron levels and significantly increased lipid peroxidation in mitochondria, producing ferroptosis [271, 328, 330]. ISCs have been reported to regulate ferroptotic cell death [331]. ISCs are located in the mitochondrial matrix and are assembled by the ISC assembly enzyme, composed of cysteine desulfurase (NFS1), small accessory protein (ISD11), and the iron chaperone, frataxin (FXN) [332]. A deficiency of ISCs increased iron loading, activating IRP‐2 and increasing the likelihood of ferroptosis [333]. Recently, it was reported that FXN controls iron homeostasis and mitochondrial activity and it was a crucial regulator of ferroptosis. The expression of FXN inhibits ISCs assembly, induces iron deprivation stress, increases the accumulation of free iron, and significantly increases erastin‐induced lipid peroxidation and ferroptosis in human fibrosarcoma HT‐1080 cells [334]. The inhibition of cysteine desulfurase NFS1, an enzyme that removes sulfur from cysteine [335], induced ferroptosis in lung adenocarcinoma cells [336]. Furthermore, the mitochondrial iron–sulfur (Fe–S) protein, CDGSH iron sulfur domain 1 (CISDs), has been reported to play a role in ferroptosis [337, 338, 339, 340]. The inhibition of CISD1 and CISD2 increased iron‐mediated mitochondrial lipid peroxidation and induced erastin and sorafenib‐induced ferroptosis, in HCC cells [337, 338, 339]. The ablation of CISD3 increased the sensitivity of HT‐1080 cancer cells to ferroptosis caused by cysteine deprivation [340]. Overall, mitochondria are a hub of cellular iron metabolism and regulate mitochondrial and cellular iron status to modulate ferroptosis.

In summary, mitochondrial lipid peroxidation can induce ferroptotic cell death, and antioxidants that target mitochondria could mitigate it. Ferroptosis is also dependent on mitochondrial energy pathways, such as the glutaminolysis–TCA cycle–ETC axis, to produce ROS. Furthermore, the MMP has a dual role, as hyperpolarization facilitates ferroptosis and disruption prevents it. In summary, mitochondrial regulation of ferroptotic cell death could be therapeutic targets for diseases associated with ferroptosis.

## 6 Mitochondria in Pyroptosis Regulation

Pyroptosis is an inflammatory programmed cell death mechanism that is activated by cytosolic receptor‐mediated recognition of pathogen‐associated molecular patterns or host‐derived damage‐associated molecular patterns (DAMPs) [341]. Pyroptosis, also known as immunogenic cell death, usually occurs in macrophages and can be characterized by cell swelling and plasma membrane rupture, causing the release of proinflammatory cytokines, interleukin (IL)‐1β and IL‐18, which elicit an inflammatory response [342]. Gasdermins have been reported to be the executioners of pyroptosis [343, 344, 345]. The human gasdermin family contains six members of pore‐forming proteins []. Gasdermin D, once cleaved at the N‐terminal domain by activated caspase‐1, oligomerizes and forms a pore [344]. This pore can penetrate the plasma membrane and disrupt the concentration and electrical gradient of sodium and potassium []. The influx of sodium ions into the cell is accompanied by excess fluid, which causes the cell volume to increase, forming fluid filled “balloons,” and ultimately cell lysis [345]. In addition, these pores can induce the release of IL‐1 β and IL‐18, which contribute to pyroptotic cell death [342].

**FIGURE 6:**
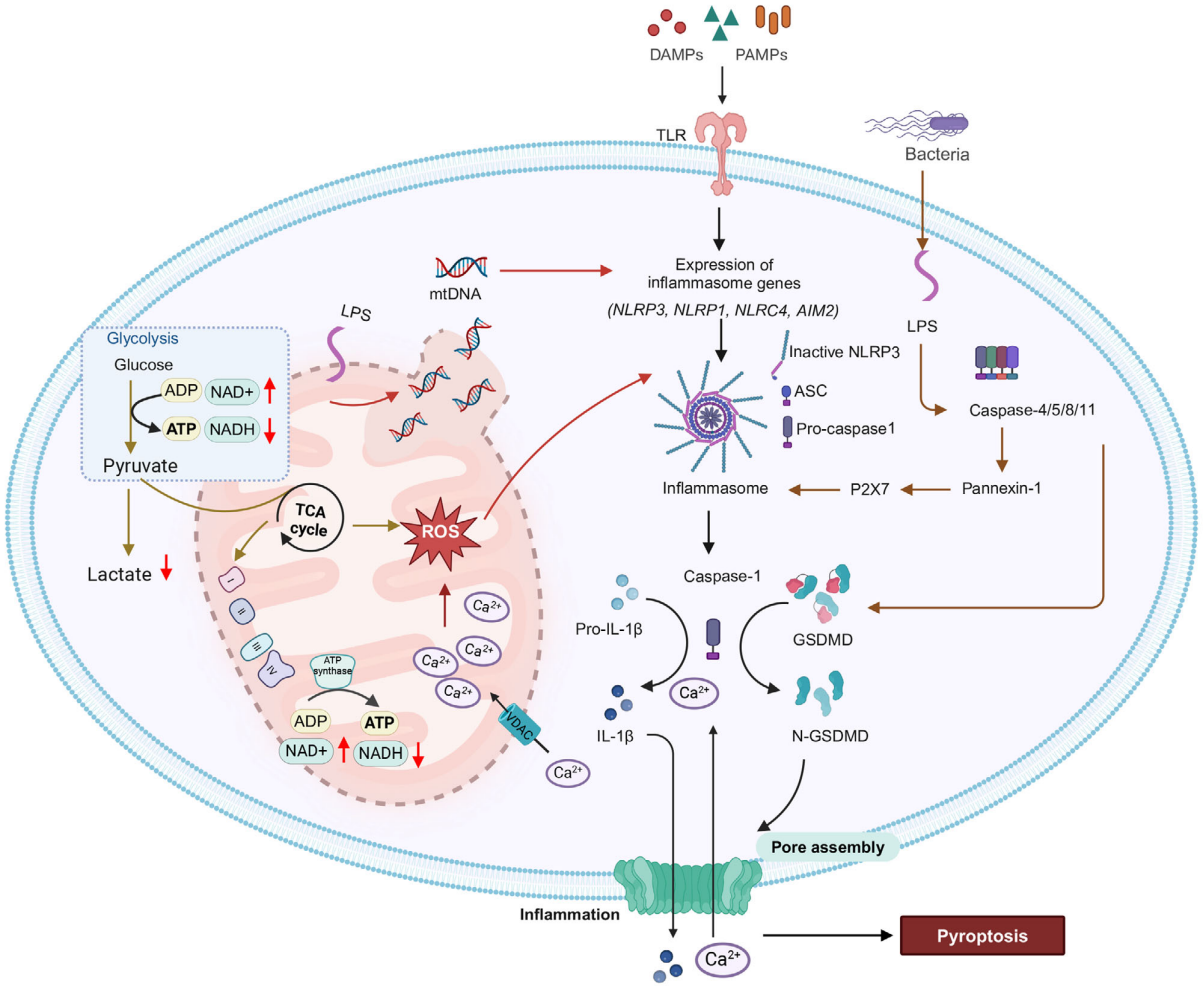
The role of mitochondria in pyroptosis. mtDNA that leaks from mitochondria is a damage‐associated molecular pattern (DAMPs) molecule that can induce the activation of the Nod‐like receptor family pyrin domain containing 3 (NLRP3) inflammasome, which further activates caspase‐1 and gasdermin‐D (GSDMD), producing pyroptosis. mtROS is also generated by disrupting glycolytic flux, which produces increased NAD+, decreased ATP production and decreased lactate secretion, which induce inflammasome signaling and activate pyroptosis. The figure created with BioRender.com.

Pyroptosis is regulated by either by a caspase‐1‐independent or caspase‐1‐dependent mechanism [346]. In the caspase‐1‐dependent mechanism, also known as the canonical pathway [], canonical inflammasomes are activated and induce pyroptosis [342]. The canonical inflammasome pathway consists of the assembly of multiprotein complexes that detect exogenous pathogens and endogenous cell damage [342]. These inflammasome detectors include or NOD‐like receptor (NLR) family, pyrin domain containing 1B (NLRP1B), NLRP3, NLR family caspase activation and recruitment domain (CARD) domain containing protein 4 (NLRC4), absent in melanoma 2 (AIM2) and pyrin, and upon activation, they recruit caspase‐1, which biotransform gasdermin D or pro‐IL‐1β and pro‐IL‐18 to biologically active molecules [346]. These events initiate pyroptosis and due to cell swelling and plasma membrane rupture, there is a rapid release of the proinflammatory cytokines, IL‐1β and IL‐18, into the extracellular space, producing an inflammatory microenvironment [342]. In the caspase‐1‐independent mechanism, also known as the noncanonical pathway, the noncanonical activation of inflammasomes occurs []. Caspase‐4, caspase‐5, and caspase‐11 bind to LPSs from Gram‐negative bacteria via their CARD, which leads to their activation. Gasdermin D is biotransformed by this process and the NLRP3 inflammasome can be activated, producing the maturation of IL‐1β and IL‐18, by a caspase‐1 dependent cleavage [346]. The role of mitochondria in regulating pyroptosis is summarized in Figure 6.

### 6.1 mtDNA Association with Pyroptosis

MtDNA, if leaked into the cytosol, is a DAMP and it can activate the NLRP3 inflammasome [348]. Mitochondrial damage produced by LPS results in the leakage of mtDNA into the cytoplasm of the cell [348]. The leaked DNA activates the NLRP3 inflammasome, which activates caspase‐1 and gasdermin D [343]. The intracellular cyclic guanosine monophosphate‐adenosine monophosphate synthase (cGAS)–STING pathway is the main signaling pathway for DNA sensing, and after the knockdown of the gasdermin D gene, it is activated, indicating the presence of a negative feedback mechanism [349]. Therefore, gasdermin D facilitates the release of mtDNA into the extracellular space to prevent the accumulation of DNA in the cytosol and the inflammatory response caused by the cGAS–STING pathway [349, 350].

### 6.2 Mitochondria‐Derived ROS Impacts Pyroptosis

mtROS can be formed from an overload of calcium ions in the mitochondria, and from mitochondrial dysfunction [351, 352]. Increased mtROS generation can activate the NLRP3 inflammasome [353]. NLRP3 activation results in lysosomal damage, which can further activate the inflammasome [341]. The upstream molecular mechanisms are still not understood but it has been hypothesized that the mitochondrial antiviral signaling protein and the serine–threonine kinase, NIMA‐related kinase 7, are involved in pyroptosis [354]. Pyroptosis activation can produce irreversible mitochondrial damage [355]. mtROS can localize around the damaged mitochondria and signal for gasdermin D oxidation [355]. Therefore, gasdermin D is the key ROS‐targeting protein during oxidative stress. Gasdermin D also contributes to the fragmentation of the mitochondrial network. In gasdermin D‐deficient models, morphological changes were not observed; however, in gasdermin D pyroptosis, there were rounded, rather than filamentous mitochondrial networks [349].

It is believed that mtROS and its relationship to pyroptosis may be linked to a negative feedback regulation between pyroptosis and mitophagy [356]. Caspase‐1 activation increases mtROS levels and mitochondria fragmentation [357]. Mitochondrial damage is increased by caspase‐1 cleaving of the protein, Parkin, a key regulator of mitophagy [357]. This biotransformation resulted in the inhibition of mitophagy, release of DAMPs, and increased plasma membrane permeabilization [357]. IL‐1β secretion in macrophages increased in tandem with the inhibition of mitophagy; however, it remains to be determined if mitophagy interferes with the biotransformation of cytokines of IL‐1 family for secretion into specialized secretory vesicles [358]. In contrast, the activation of mitophagy can remove damaged mitochondria, which decreases the magnitude of inflammasome‐mediated effects [359].

In summary, mitochondria play an important role in regulating pyroptosis. For example, as a result of mtDNA release, ROS production, and/or interactions with inflammasome components. The release of mtDNA into the cytosol acts as a DAMP that activates the NLRP3 inflammasome and gasdermin D, releasing proinflammatory cytokines, inducing pyroptosis. It has also been shown that mitochondrial dysfunction also influences pyroptosis through a complex feedback loop with mitophagy. Although pyroptosis inhibits mitophagy, increasing inflammation, mitophagy can attenuate pyroptosis by clearing damaged mitochondria and limiting inflammasome activation. These insights provide information about mitochondria as a key mediator of inflammation through pyroptosis, highlighting them as a potential target for inflammatory diseases.

## 7 Mitochondria in Paraptosis Regulation

**FIGURE 7:**
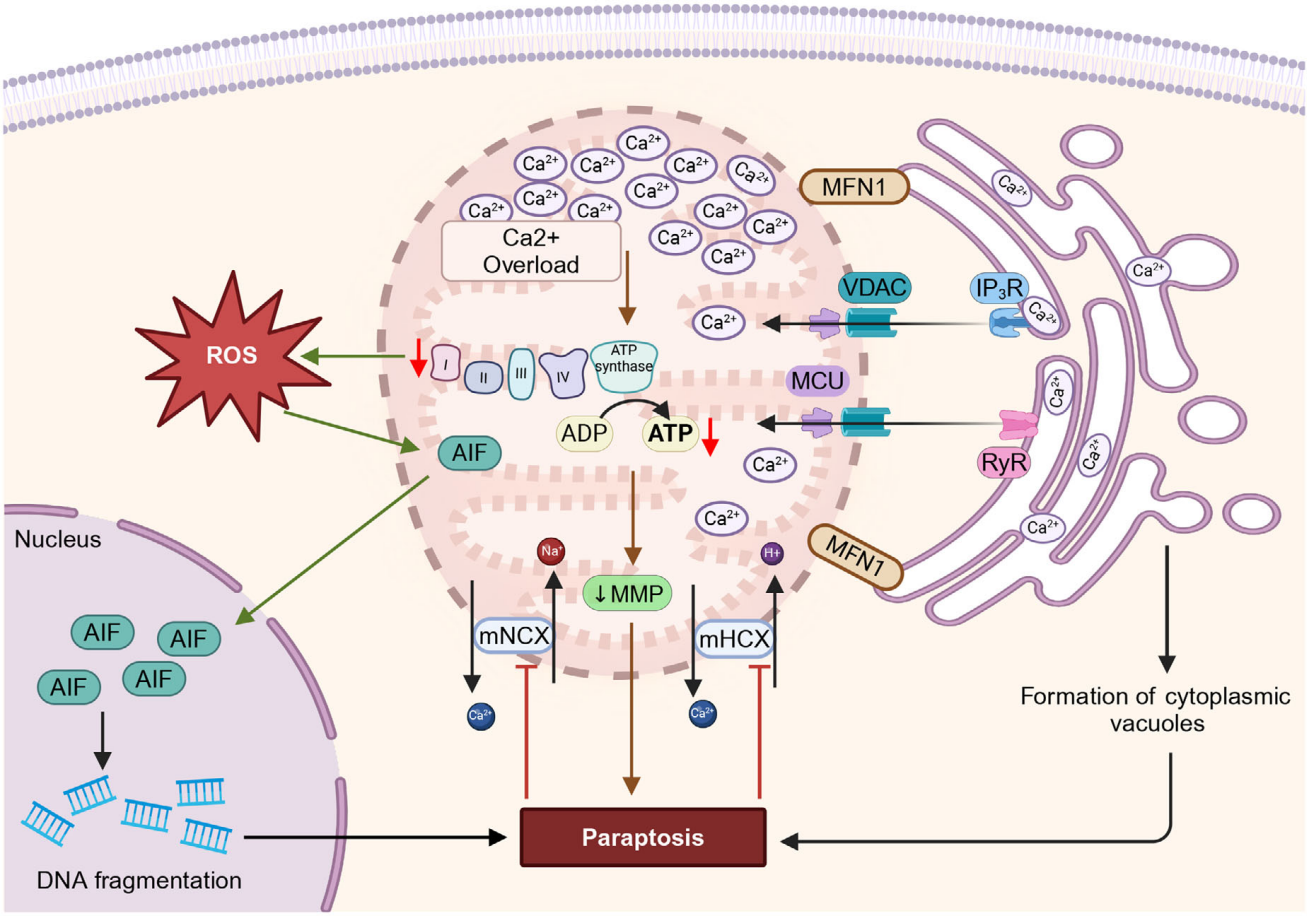
The role of mitochondria in paraptosis. The influx of Ca^2+^ ions occurs from the ER to the mitochondria due to the activation of inositol 1,4,5‐triphosphate receptors (IP_3_Rs) and ryanodine receptors (RYRs) in the mitochondria. Ca^2+^ overload results in decreased ATP production and a loss of mitochondrial membrane potential, which ultimately causes paraptosis. Paraptosis involves mitochondrial dysfunction that is mediated by inhibition of ETC complex I and decreased ATP synthesis, which is followed by ROS generation, translocation of apoptosis‐inducing factor (AIF) from mitochondria to the nucleus, DNA fragmentation and cell death due to paraptosis. The figure created with BioRender.com.

Paraptosis is a regulated NACD that is characterized by cytoplasmic vacuolization involving the swelling of the ER and mitochondria [360, 361]. Paraptosis lacks apoptotic morphological features (e.g., nuclear condensation and fragmentation, cell membrane blebbing, formation of apoptotic bodies, and loss of ribosome from cisternae) [362] and cells undergoing paraptosis appear round, vacuolated, and swollen [363]. This swollen morphology may indicate intracellular ion dysregulation, which is followed by water retention, eventual osmotic lysis, and release of intracellular contents [364]. Intracellular contents, such as ATP, uridine tri phosphate, high‐mobility group protein B1 (HMGB1), heat‐shock proteins, and various proteases, act as “danger signals” [360], leading to extensive inflammation and cell‐mediated immunity [360]. Although the molecular mechanisms of apoptosis are well established, many aspects of paraptosis remain to be determined. However, disruption in cellular proteostasis (due to proteasomal inhibition or protein thiol homeostasis disruption) and ion homeostasis (e.g., Ca^2+^, and K+), which trigger stress to ER and mitochondria, appear to play an important role in paraptosis [365, 366]. Paraptosis was initially reported by Sperandio et al. [367] in 293T, epithelial‐like kidney cells, overexpressing insulin‐like growth 1 receptor, which induced paraptosis through the activation of two signaling pathways: MAPK and C‐Jun N‐terminal kinase [368]. The study also reported that the apoptosis‐linked gene 2 interacting protein, Alix, inhibited paraptosis [368]. Hoa et al. [360] investigated the mechanism of paraptosis induction by monocytes in human glioma cells. The results indicated that osmotic dysregulation in tumor cells induced by the disruption of ionic homeostasis and the activation of big potassium (BK) channels [368], which are located in ER and mitochondria, could explain why these two organelles are specifically targeted in paraptosis. Ca^2+^‐mediated interaction between the ER and mitochondria is essential for paraptosis since ER and mitochondria also store Ca^2+^ ions [360, 361]. The intracellular Ca^2+^‐flux system located at the ER–mitochondrial axis can cause mitochondrial dilation during paraptosis, whereas the accumulation of misfolded proteins within the ER lumen have been postulated to exert an osmotic force and draw water from the cytoplasm, producing distension of the ER lumen [369]. Jambrina et al. [370] reported that Ca^2+^ overload produced mitochondrial damage, producing paraptotic cell death in Jurkat cells. Since BK channels are Ca^2+^ activated voltage‐dependent K channels, which are electrically activated, or by increasing Ca^2+^ flux the findings of Hoa et al. [360] are similar to those of Jambrina et al. [370]. A variety of natural products and chemicals cause paraptosis cell death by depleting ER Ca2^+^ and overloading mitochondrial Ca^2+^. The role of mitochondria in regulating paraptosis is summarized in Figure 7.

### 7.1 Mitochondrial Dysfunction in Paraptosis

In paraptosis, early‐stage mitochondrial swelling is thought to be an adaptive response that allows for the removal of calcium released by the ER, and the maintenance of mitochondrial function, to postpone cell death [371]. During proteasomal inhibition and an excess of calcium in the mitochondria, vacuoles are formed from the ER and the mitochondria [372, 373]. Due to the swelling and extensive cytoplasmic vacuolization that occurs during paraptosis, inducing paraptosis could be used as a therapeutic strategy to kill cancer cells that are resistant to apoptosis [374]. Ca^2+^ ions released from the ER by ryanodine receptors (RYRs) and inositol IP_3_Rs, flow across the OMM, primarily via the VDAC [375]. Ca^2+^ ions enter the intermembranous space and pass through the IMM, primarily via the mitochondrial Ca^2+^ uniporter (MCU) complex, which allows Ca^2+^ flux into the mitochondria, resulting in Ca^2+^ overload [376]. The Na^+^/Ca^2+^ exchanger in mitochondria are also inhibited by Ca^2+^ overload [377, 378]. Subsequently, the MMP is lost, leading to proton accumulation [377, 378]. Mitochondrial and ER swelling occur, which is caused by mitochondrial and ER dilation [225]. Ginger extract induces paraptosis in MDA‐MB‐231 and A549 cancer cells by producing mitochondrial dysfunction, characterized by a loss of the MMP, decreased ATP production, increased ROS levels and DNA fragmentation, due to the translocation of apoptosis‐inducing factor (AIF) from mitochondria to the nucleus [379].

### 7.2 Mitochondrial Calcium Overload in Paraptosis

Natural and chemically induced paraptosis‐mediated vacuolization occurs through the dilation of mitochondria and ER, by producing calcium overload in the mitochondria, leading to cell death [365, 378]. Yoon et al.[369] reported that curcumin (a natural polyphenol isolated from rhizomes of *Curcuma longa* [380]), induced paraptosis, by inducing the release of Ca^2+^ from ER, in tandem with MCU‐mediated mitochondrial Ca^2+^ uptake, leading to mitochondrial Ca^2+^ overload and subsequent dilation of mitochondria and ER in the breast cancer cell lines, MDA‐MB‐435S and MDA‐MB‐231 [373, 381]. Yoon et al. later reported that celastrol, a compound present in *Tripterygium wilfordii* [382, 383], at 1.2, 0.5, and 4 µM, caused the IP_3_R‐mediated release of Ca^2+^ from the ER and MCU‐mediated mitochondrial Ca^2+^ uptake, producing paraptosis in MDA‐MB‐435S cells [369]. IP_3_R and RyR mediated the release of Ca^2+^ from ER and its MCU‐mediated mitochondrial Ca^2+^ uptake, contributing to hesperidin (a flavanone glycoside present in many citrus fruits and vegetables [384, 385])‐induced paraptosis in human hepatoblastoma, HepG2 cells [386]. Morusin, a prenylated flavonoid isolated from *Morus australis* root bark, induced paraptosis, at 30 µM, through VDAC‐mediated mitochondrial Ca^2+^ overload and MMP (ΔΨM) depletion, in the human epithelial ovarian cancer cells, A2780 and SKOV‐3 [387]. Fontana et al. [388] reported that mitochondrial Ca^2+^ overload is required for δ‐tocotrienol‐mediated paraptosis in the castration resistance prostate cancer cells, PC‐3 and DU145. In addition to Ca^2+^ overload, δ‐tocotrienol also induced mitochondrial dysfunction in A375 and BLM cell lines, by inhibiting the expression of the OXPHOS complex I, producing an accumulation of ROS, which played a role in inducing paraptosis [389]. Chalcomoracin, a major secondary metabolite found in fungus‐infected mulberry leaves, at 6 µM, abolished the MMP and disrupted Ca^2+^ homeostasis, producing paraptosis cell death in MDA‐MB‐231 and PC‐3 cancer cells [390]. The incubation of MCF‐7 and MDA‐MB‐231 cancer cells with 4 µM of withaferin‐A, isolated from the root of *Withania somnifera* Dunal [391], caused paraptosis that was characterized by swollen mitochondria associated with hyperpolarization of the mitochondrial membrane [392]. The incubation of human lung carcinoma, ASTC‐a‐1 cells, with 70 µM of taxol, an anticancer compound [393], produced the loss of the MMP before the formation of vacuoles and the collapse of the microtubule cytoskeleton [394]. Interestingly, a cyclometalated iridium (Ir) complex‐peptide hybrid and CGP37157, an inhibitor of a mitochondrial sodium (Na^+^)/Ca^2+^ exchanger [395], induced paraptosis by mitochondria and ER membrane fusion or by tethering via the MAMs, which allowed the transport of Ca^2+^ transport between ER to mitochondria, producing a decrease in ΔΨM and subsequent paraptosis death in Jurkat cells [396].

In summary, paraptosis occurs when calcium overload and disruption of ion homeostasis are mediated by mitochondria. This results in ER swelling and mitochondrial damage, producing cell death. During paraptosis, cellular features such as vacuolation and mitochondrial dilation occur, which differ from classical apoptosis. The accumulation of calcium in mitochondria can produce changes in membrane potential, which are frequently induced by natural compounds or chemical inducers. In addition to swelling, calcium overload results in increased ROS levels, which drive paraptotic cell death.

## 8 Mitochondria in Parthanatos Regulation

**FIGURE 8:**
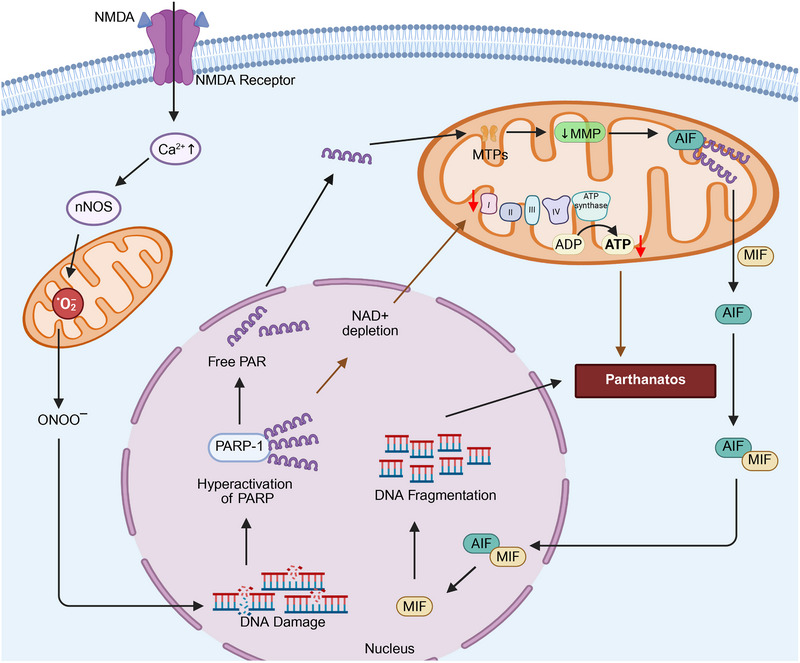
The role of mitochondria in parthanatos. Excessive DNA damage by free radicals, ionizing radiation, hydroxy radicals, and activation of NMDA receptors, causes the overactivation of poly (ADP‐ribose) polymerase‐1 (PARP‐1). Free PAR polymers induce the opening of mitochondrial permeability transition pores (MPTP), which results in the loss of the mitochondrial membrane potential. This induces the translocation of AIF from mitochondria to the nucleus, where it binds to the protein, macrophage migration inhibitory factor (MIF), which further results in DNA fragmentation and activates the mitochondria. PARP over activation also produce a depletion of NAD+, resulting in decreased ATP production by inhibiting complex I of the ETC chain, inducing parthanatos. The figure created with BioRender.com.

Parthanatos is a highly regulated, poly (ADP‐ribose) polymerase‐1 (PARP‐1)–dependent mechanism of cell death first discovered by Yu et al. in 2002 [397] and named as such in 2008 [398]. The word parthanatos is a portmanteau of the words “par” (from poly ADP ribose polymer, formed after PARP‐1 activation) and “Thanatos,” the personification of the Greek god of death [399, 400]. Since the discovery of the nuclear enzyme, PARP‐1, the foundational member of the PARP family, over 50 years ago [401], 17 PARPs have been characterized [402]. PARP‐1 has a number of biological functions, including the maintenance of the genome and repair of DNA and acting as a DNA damage sensor [403, 404, 405] and regulating other metabolic and homeostatic processes, such as cell death [406, 407, 408], gene transcription [409, 410], protein turnover, and inflammation [411]. PARP‐1's role in tumor progression has been thoroughly characterized [412, 413], which led to the development of PARP inhibitors for the treatment of various types of cancer [414, 415]. The role of PARP inhibitors for the treatment of oncological diseases has been investigated and it has been reported that PARP‐1 inhibitors may be efficacious in the management of myocardial infarction, cardiopulmonary bypass, and chronic heart failure [416, 417, 418]. It is important to state that PARP‐1 is also known as poly(ADP‐ribose) synthetase or poly(ADP‐ribose) transferase [419]. PARP‐1 cleaves NAD^+^ to form ADP‐ribose and nicotinamide and catalyzes the attachments of ADP‐ribose to substrate proteins, a posttranslation modification known as PARylation [401]. PARP‐1 can cleave additional molecules of NAD^+^ and several ADP‐ribose residues can be attached to the same protein, forming poly(ADP‐Ribose) (PAR) chains [420]. The synthesis of these PAR chains and their removal and cleavage by poly(ADP‐ribose) glycohydrolase and ADP‐ribosyl hydrolase 3, are enzymatic processes present in virtually all eukaryotic cells [420]. Thus, PARP enzymes and specifically, PARP‐1, produce their regulatory functions primarily by directly regulating PARylation and cellular NAD^+^ stores. The role of mitochondria in regulating parthanatos is summarized in Figure 8.

**Mitochondrial Dynamics and the Molecular Basis of Parthanatos**: The early phase of parthanatos is initiated by excessive DNA damage, produced by hydrogen peroxide, free radicals, ionizing radiation, and hydroxy radicals [421]. The activation of N‐methyl‐D‐aspartate (NMDA) receptor, can also produce DNA damage by increasing the cellular influx of calcium, activation of nitric oxide synthase, followed by an increase in ROS levels [421]. DNA damage produces rapid and robust activation of PARP‐1, the synthesis and accumulation of PAR polymers, alteration of the MMP, and nuclear translocation of mitochondrial AIF [422]. AIF translocation is considered to be the biological process that subsequently produces parthanatos [398]. In a later stage of parthanatos, caspases are activated; however, caspase inhibition does not protect cells from parthanatos [423], indicating that caspase activation is a secondary occurrence and not a main process that produces cell death after the initiation of parthanotos [398]. In contrast, during apoptosis, PARP‐1 and other PARP‐related enzymes are cleaved and inactivated by caspases, possibly to conserve ATP, since apoptosis is an ATP–dependent process and PARP‐induced cell death depletes ATP levels [424, 425]. Moreover, PARP inhibition can induce apoptosis of cells that were likely to undergo necrosis [424, 425].

The hyperactivation of PARP‐1 depletes NAD^+^ stores, producing  mitochondrial dysfunction and decreased ATP production, as well as changes that occur during parthanatos, that ultimately cause cell death [398, 420, 426]. After DNA damage, the overactivation of PARP‐1 has a significant effect on mitochondrial oxidative metabolism, characterized by: (1) the loss of the MMP; (2) decreased mitochondrial oxygen consumption [427]; (3) decreased activity of complex I of the ETC [428]; (4) decreased activity of NADH oxidase and NADH Q1‐oxidase [429]; (5) decreased mitochondrial ATP production [430]; (6) increased levels of superoxide radicals [431]; and (7) disruption of the mitochondrial architecture [430]. Mitochondrial architectural changes included swelling and alterations of the IMM trabecular system [431]. This is followed by opening of mPTPs, which then release CL, AIF, cytochrome *c*, and caspases [397, 431]. The regulation of mitochondrial quality is impaired after PARP activation and the overactivation of PARP produces defective mitophagy [432], thereby decreasing the efficiency of the removal of damaged mitochondria. Cells undergoing parthanatos have morphological changes similar to necrosis and apoptosis [433], such as loss of cell membrane integrity, positive propidium iodide staining, externalization of phosphatidylserine(PS), chromatin condensation, DNA fragmentation [398, 434, 435], nuclear shrinkage and chromatin [398]. These morphological features occur in the presence of the mitochondrial changes. PAR accumulation, secondary to PARP‐1 overactivation, is another key signaling step parthanatotic‐induced cells death. Toxic levels of PAR translocate from the nucleus to the cytosol, where it signals AIF, by PARylation, to translocate from the mitochondria to the nucleus [435]. When AIF is no longer in the mitochondria, it recruits and interacts with macrophage migration inhibitory factor (MIF) and forms the AIF/MIF complex in the cytoplasm [436]. The translocation of the complex to the nucleus results in DNA fragmentation and chromatin condensation, ultimately producing cell death [436].

In conclusion, mitochondria play a significant role in parthanatos by contributing to cellular energy depletion, oxidative stress, and DNA damage responses. When DNA damage is extensive, PARP‐1 is activated, causing mitochondrial dysfunction and ATP reduction, resulting in the release of AIF, producing DNA fragmentation and chromatin condensation, that are hallmarks of parthanatos. Considering mitochondria's role in PARP‐1‐regulated processes and AIF signaling, it is apparent that mitochondria are crucial to parthanatos, which could be a potential therapeutic target.

## 9 Mitochondria in Cuproptosis Regulation

Copper (Cu) is essential for mitochondrial respiration and enzyme activity. Extracellulalry, Cu primarily exists as Cu^2+^, which is reduced to Cu⁺ on the cell surface by metalloreductases, such as STEAP. Cu^+^ enters the intestinal tract and other organelle cells, primarily through specific transporters known as SLC31A1 (CTR1) and SLC31A2 (CTR2). CTR1 is a high‐affinity plasma membrane protein is essential for mammalian development and uptake and maintenance of Cu levels from dietary Cu [437, 438]. Following absorption, Cu^+^ is transported to the mitochondrial matrix via the solute carrier family 25‐member 3 (SLC25A3) receptor [439]. Cu functions as a necessary cofactor within COX, facilitating electron transfer from cytochrome *c* toward oxygen, which is involved in the proton motive force [440]. Cu also plays a pivotal role in cellular defense against oxidative stress through Cu‐containing SOD (Cu/Zn SOD). Under conditions of low Cu exposure or short exposure durations, Cu is primarily sequestered in the mitochondria to maintain homeostasis. Cu plays a dual role in tumor development. Although moderate levels can facilitate tumor growth by ROS, increasing genetic mutations (genomic instability), and influencing cellular signaling pathways, excessively high Cu concentrations can induce tumor cell death if they surpass a certain limit [439, 441].

**FIGURE 9:**
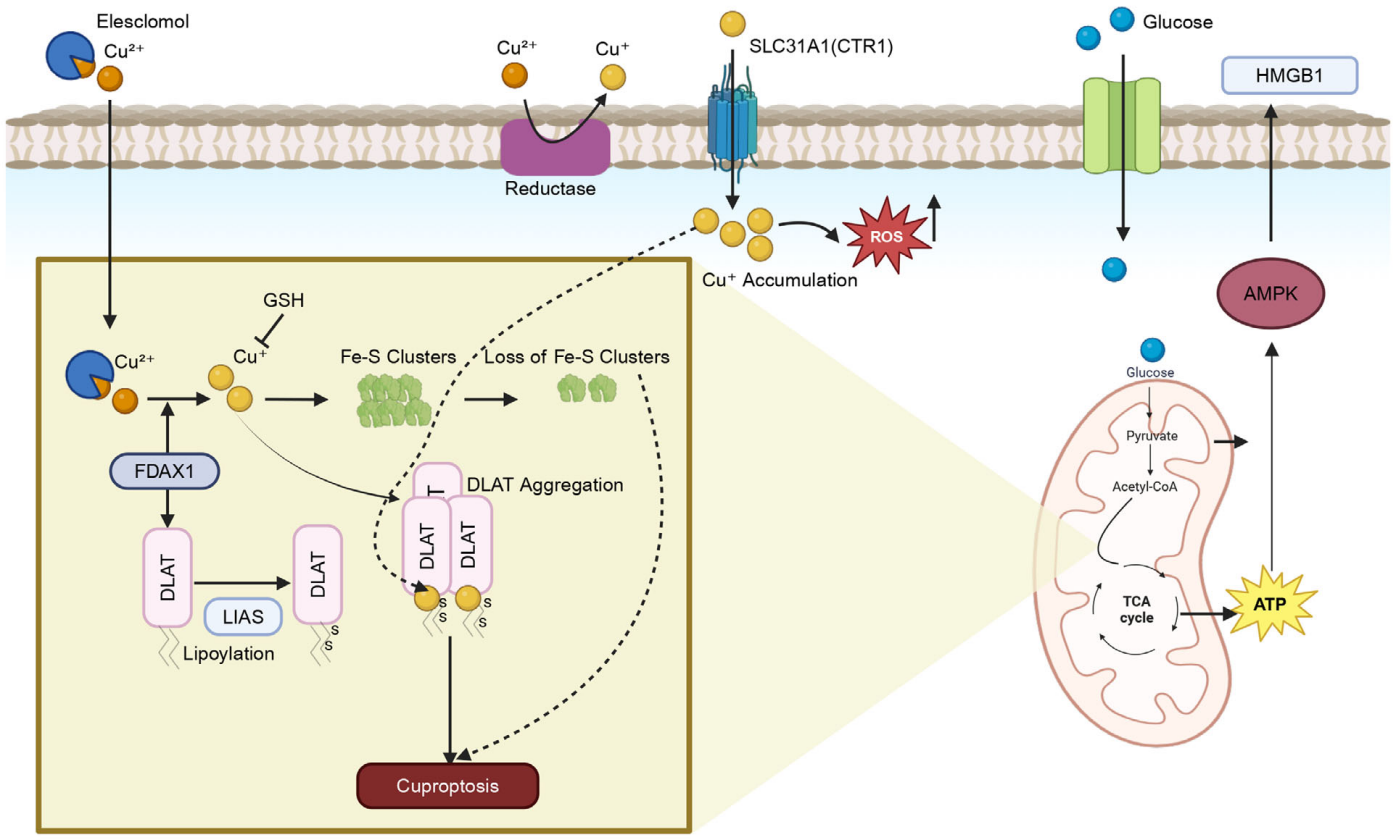
The role of mitochondria in cuproptosis. It is initiated by elevated copper (Cu) levels and involves a series of molecular mechanisms. Elesclomol facilitates the binding of Cu ions (Cu^2+^) in the extracellular region and subsequently transports them intracellularly. Once inside, reductase enzymes convert Cu^2+^ to Cu^+^, allowing cellular entry. Cu importers, SLC31A1, modulates intracellular Cu^+^ concentrations. The buildup of Cu within cells leads to an increase in reactive oxygen species (ROS). Furthermore, the enzyme FDX1 reduces Cu^2+^ to Cu^+^, enhancing the lipidation and aggregation of DLAT enzymes, which are important in regulating the mitochondrial TCA cycle. FDX1 also induces destabilization of Fe–S cluster proteins, influencing cellular sensitivity to Cu‐induced apoptosis. The thiol‐containing copper chelator, glutathione (GSH), acts to inhibit cuproptosis. Copper‐induced damage to the mitochondrial respiratory chain results in the hyperactivation of the energy sensor AMPK, which increases cuproptosis and causes the release of the proinflammatory mediator HMGB1, a key player in the inflammatory response. The figure created with BioRender.com.

**Mitochondrial Dynamics and the Molecular Basis of Cuproptosis**: Cuproptosis is a mitochondrial cell death ignited by Cu [438]. Cells that are dependent on mitochondrial respiration are more susceptible to Cu‐induced cell death [442]. A hallmark of cuproptosis is the aggregation of lipoylated mitochondrial enzymes, such as dihydrolipoamide S‐acetyltransferase (DLAT), along with a depletion of Fe–S cluster proteins. The Tsvetkov group discovered a new type of cell death triggered by the abnormal buildup of Cu in mitochondria starting a regulated cell death process called cuproptosis [443]. As previously noted, Cu is vital for maintaining homeostasis and acts as a cofactor for several enzymes involved in ATP synthesis within mitochondria [444]. The Cu ionophore elesclomol has been a focus of recent research, since it is believed to be crucial of finding out how to induce cuproptosis. Elesclomol binds to Cu^2+^ in the extracellular environment and transports it into the mitochondrial matrix [445]. Excess Cu is brought into cells through the Cu transporter CTR1, producing a significant = accumulation of intracellular superoxides [445]. Within the matrix, Cu^2+^ is reduced to Cu^+^ by Ferredoxin 1 (FDX1) [444]. FDX1 is an essential enzyme in the cuproptosis pathway, due to its potent reducing capability. It facilitates the lipoylation of four specific mitochondrial enzymes: DLAT, dihydrolipoamide S‐succinyltransferase (DLST), glycine cleavage system protein H (GCSH), and dihydrolipoamide branched chain transacylase E2 (DBT). Lipoylation is a process that occurs when a small molecule, lipoic acid attaches itself to specific enzymes, increasing their activity. Moreover, FDX1 is an upstream regulator of protein lipoylation. The absence of FDX1 disrupts protein lipoylation [440, 444]. FDX1 has two primary roles: it interacts with lipoyl synthase to catalyse the lipoylation of DLAT, which regulates the mitochondrial TCA cycle, and it destabilizes Fe–S cluster proteins. Additionally, Cu‐induced damage to the mitochondrial respiratory chain leads to hyperactivation of the energy sensor, AMPK, increasing cuproptosis and triggering the release of high mobility group box 1 (HMGB1), a mediator of sterile inflammation resulting from cuproptotic injury [442]. Cu^+^ demonstrably enhancsignificantly increases protein aggregation and this is due to to their interaction with protein structures, which disrupts the intricate folding pathways essential for proper function. Cu^+^ binding can expose hydrophobic regions within the protein, facilitating intermolecular interactions and producing protein aggregation [439, 446]. GSH also plays a crucial role in modulating cuproptosis [447]. GSH can bind to to Cu⁺ via its thiol group, thereby mitigating Cu's cytotoxic effects. GSH prevents the toxicity of ES–Cu by chelating intracellular Cu, highlighting GSH's protective role against cuproptosis [438, 448]. The involvement of mitochondria in Cuproptosis is summarized in Figure 9

## 10 Conclusion and Perspective

Mitochondria, the powerhouse of the cell, are highly dynamic organelles that have long been linked to the production of ATP and metabolism in cells, but they are now recognized as supporting a wide range of other functions. It has been well established that mitochondria are key to regulating apoptosis. However, increasing data indicate that they are also involved in NACD unrelated to classic apoptosis. This article describes the mechanisms underlying NACD, as well as mitochondria's specific role in initiating or facilitating the death response. Evidence suggests mitochondrial components play a role in NACD, including necroptosis, pyroptosis, autophagy, ferroptosis, paraptosis, parthanatos, and cuproptosis, although their significance may vary.

As an example, mitochondria affect necroptotic cell death by altering mitochondrial membrane permeability, which is regulated by Cyp‐D and PGAM5, which are downstream of RIPK1–RIPK3–MLKL. Although ROS regulate necroptosis differently in different cells, BCL‐2 proteins such as BMF, MAX, BAK, and BNIP3 affect necroptosis susceptibility. During autophagy, mitochondria serve as activation sites for the formation of autophagosomes as well as providing membranes. Through NACD in autophagy, damaged mitochondria are disposed of through mitophagy, which preserves cellular stability. Furthermore, mitochondrial oxidative stress and TCA cycle molecules, such as glutamine, a‐KG, GLUD1, and UQCRC1, regulate autophagy. Oxidative stress is induced by the increase in mitochondrial lipid peroxidation, resulting in ferroptosis. Similarly autophagy, ferroptosis is regulated by transaminases and TCA components, such as the glutaminolysis‐regulating genes,  *SLC38A1, SLC1B5*, and *GLS2*. Calcium overload in the mitochondria facilitates paraptosis, resulting in dilation of the mitochondria and ER, resulting in vacuolization of the cytoplasm. In contrast excessive PARP‐1 activation causes parthanatos, leading to NAD+ depletion and mitochondrial dysfunction by inhibiting ETC complex I. Mitochondrial AIF also has a significant role in parthanatos induction, as it translocates to the nucleus and binds with MIF, causing DNA fragmentation and cell death. However, increased levels of intracellular Cu induces cuproptotic cell death through FDX1‐regulated proteotoxic stress. FDX1 enhances the lipoylation and accumulation of mitochondrial enzymes such as DLAT, DLST, GCSH, and DBT and destabilizes Fe–S cluster proteins, which leads to cell death.

The majority of these NACDs are regulated differently from classical apoptosis but still share the involvement of mitochondria through mtROS production, mitochondrial membrane disruption, or metabolic changes. The involvement of mitochondria in necroptosis is highly cell type specific. In necroptosis, mtROS generation results in mitochondrial membrane disruption, hindered ATP production. Autophagy involves the recycling of damaged mitochondria to maintain homeostasis, however, excessive mitophagy diminish mitochondrial content and elevate the burden on other organelles triggering cell death. Furthermore, in autophagy, mitochondria regulate energy sensing pathways such as, AMPK and mTOR and in response to a decrease mitochondrial ATP production, autophagy is induced in an mTOR/AMPK dependent manner. Iron metabolism in the mitochondria leads to mtROS, which increases lipid peroxidation and facilitate iron‐dependent ferroptosis when mitochondria are hyperpolarized. The mitochondria also play an important role in pyroptosis. Specific mtDNA leakages facilitate the activation of the inflammasome. Pyroptosis is further propagated by alteration of mitochondrial metabolism and generation of mtROS. Furthermore, there are parallels between parthanatos and paraptosis in which mitochondria play a crucial role. In paraptosis, Ca^2+^ overload in mitochondria causes mitochondrial dysfunction, resulting in DNA fragmentation and cell death. Similarly, there are some notable commonalities in the role of mitochondria in paraptosis and parthanatos. In paraptosis, overload of calcium in mitochondria increases for cellular ROS generation and mitochondrial dysfunction that facilitates AIF‐mediated DNA fragmentation and cell death. However, in parthanatos, overactivation of PARP, due to DNA damage not only leads to mitochondrial dysfunction, which is followed by loss of MMP and decrease in ATP, but also the translocation of mitochondrial AIF to nucleus to promote further DNA fragmentation that increases cell death. In contrast, in cuprotosis, damage to the mitochondrial respiratory chain results in overactivation of the energy sensor, AMPK, which releases proinflammatory molecules HMGB1, inducing cell death. Although mitochondrial contribution in these cell death processes overlap; studies indicate their specific roles differ in each pathway. Autophagy, for example, relies on mitochondria, while necroptosis paired with ferroptosis has a strong mtROS connection. Detailed knowledge of these intricate roles can provide a guide to develop therapeutic stratergies.As a result of alterations in mitochondrial functions, these strategies can also be applied to cancer, where they may contribute to NACD.

Mitochondria play a central regulatory role in NACD pathways, underscoring the importance of mitochondrial‐targeted therapies. Investigating the interplay between mitochondrial alterations—particularly in energetics, biogenesis, and dynamics—and NACD is essential for developing effective cancer treatments. Ongoing clinical trials are evaluating mitochondrial‐targeting agents like MitoTam, a tamoxifen derivative that accumulates in mitochondria. Although its exact mechanism remains unclear, MitoTam disrupts mitochondrial respiration by targeting cytochrome P450 enzymes and shows potential in overcoming apoptotic drug resistance [449]. There is increasing interest in leveraging autophagy to treat cancers. The combination of autophagy selective therapeutics—such as metformin, rapamycin, and dasatinib (autophagy inducers), with hydroxychloroquine and nelfinavir (autophagy inhibitors)—is in phase I/II clinical trials for advanced solid tumors and relapsed prostate cancers. Similarly, the autophagy‐inducing agent ABTL0812 is undergoing phase IIb/III trials, combined with chemotherapeutics (leucovorin calcium, fluorouracil, irinotecan, and oxaliplatin) as a first‐line treatment for advanced pancreatic cancer. Although mitochondria do not directly initiate ABTL0812‐induced cell death, its mechanism involves mitochondrial depolarization and disruption [450]. These findings, along with continued research, will prove invaluable for the development of mitochondria‐targeted therapies capable of disrupting cancer proliferation, altering metabolism and triggering NACD.

## Author Contributions

Conceptualization: S. M. and A. K. T. Software: S. S., N. H., and S. M. Validation and formal analysis: R. J. B., C. R. A., and A. K. T. Resources: R. J. B. and A.K.T. Data and figure curation: S. M., A. K. T., D. T., S. S., and R. N. Writing—original draft preparation: S. M., N. H., R. N., M. A. D., and C. R. A. Writing—review and editing: D. T., S. S., R. J. B., and C. R. A. Visualization: S. M. and S. S. Supervision: A. K. T. and R. J. B. Project administration: A. K. T. Funding acquisition: A. K. T. All authors have read and agreed to the published version of the manuscript.

## Ethics Statement

This article is a review of existing literature and does not involve any original research with human participants or animals. Therefore, ethical approval was not required for this study.

## Conflicts of Interest

The authors declare no conflicts of interest.

## Data Availability

The data and materials referenced in this review article are publicly available and accessible in accordance with the journal's data sharing policy. The authors have no objections to the availability or use of these data. Further inquiries can be directed to the corresponding author A. K. T.
